# Microarray analysis reveals ONC201 mediated differential mechanisms of CHOP gene regulation in metastatic and nonmetastatic colorectal cancer cells

**DOI:** 10.1038/s41598-021-91092-8

**Published:** 2021-06-04

**Authors:** Ashraf Al Madhoun, Dania Haddad, Mustafa Al Tarrah, Sindhu Jacob, Waleed Al-Ali, Rasheeba Nizam, Lavina Miranda, Fatema Al-Rashed, Sardar Sindhu, Rasheed Ahmad, Milad S. Bitar, Fahd Al-Mulla

**Affiliations:** 1 Department of Genetics and Bioinformatics, Dasman Diabetes Institute, 15462 Dasman, Kuwait; 2 Department of Animal and Imaging Core Facilities, Dasman Diabetes Institute, 15462 Dasman, Kuwait; 3 Department of Pharmacology and Toxicology, Faculty of Medicine, Kuwait University, 046302 Jabriya, Kuwait; 4 Department of Immunology and Microbiology, Dasman Diabetes Institute, 15462 Dasman, Kuwait

**Keywords:** Cancer therapy, Cancer microenvironment, Cancer, Colorectal cancer

## Abstract

The imipramine ONC201 has antiproliferative effects in several cancer cell types and activates integrated stress response pathway associated with the induction of Damage Inducible Transcript 3 (DDIT3, also known as C/EBP homologous protein or CHOP). We investigated the signaling pathways through which ONC201/CHOP crosstalk is regulated in ONC201-treated nonmetastatic and metastatic cancer cell lines (Dukes' type B colorectal adenocarcinoma nonmetastatic SW480 and metastatic LS-174T cells, respectively). Cell proliferation and apoptosis were evaluated by MTT assays and flow cytometry, gene expression was assessed by Affymetrix microarray, signaling pathway perturbations were assessed in silico, and key regulatory proteins were validated by Western blotting. Unlike LS-174T cells, SW480 cells were resistant to ONC201 treatment; Gene Ontology analysis of differentially expressed genes showed that cellular responsiveness to ONC201 treatment also differed substantially. In both ONC201-treated cell lines, CHOP expression was upregulated; however, its upstream regulatory mechanisms were perturbed. Although, PERK, ATF6 and IRE1 ER-stress pathways upregulated CHOP in both cell types, the Bak/Bax pathway regulated CHOP only LS-174T cells. Additionally, CHOP RNA splicing profiles varied between cell lines; these were further modified by ONC201 treatment. In conclusion, we delineated the signaling mechanisms by which CHOP expression is regulated in ONC201-treated non-metastatic and metastatic colorectal cell lines. The observed differences could be related to cellular plasticity and metabolic reprogramming, nevertheless, detailed mechanistic studies are required for further validations.

## Introduction

Imipridones are a class of small molecules with anticancer properties. The founding member TRAIL-inducing compound 10 (TIC10), also known as ONC201, exhibits attractive physical and biochemical characteristics with selectivity toward a broad range of tumor cells but not normal cells (best reviewed by Allen et al.^1^). Currently, ONC201 is under evaluation in advanced clinical trials for a number of solid tumor malignancies including gliomas, recurrent or metastatic endometrial carcinoma, recurrent ovarian and peritoneal cancers, refractory metastatic breast cancer, relapsed non-Hodgkin’s lymphoma, and advanced neuroendocrine tumors^2^. A phase I clinical study confirmed the following: the compound is well tolerated, evidence of functionality after oral administration exists, the desired micromolar plasma concentration for advanced cancer patients is indicated, and patients exhibit an enhanced immune response^3^.

Using a human colorectal cancer cell line, ONC201 was identified while screening small molecules that induce tumor necrosis factor (TNF)-related apoptosis-inducing ligand (TRAIL) gene expression^4^. TRAIL is an attractive antiproliferative agent because it can induce apoptosis, mainly in tumor cells, by activating death receptors 4 and 5 (DR4 and DR5), while exhibiting minor toxicity against normal cells^5,6^. In solid tumor cells, ONC201 activates activating transcription factor 4 (ATF4), which is a hallmark of the integrated stress response^7^. This causes a dual alpha serine/threonine-protein kinase/ extracellular signal-regulated kinase (Akt/ERK) inactivation and the subsequent activation of the transcription factor forkhead box O3a (FOXO3a), which upregulates TRAIL gene expression^4^. In hematologic malignancies, ONC201 inhibits the Akt/inhibitor of apoptosis protein (IAP) pathway, downregulates the antiapoptotic proteins Bcl2 and Bcl-xl and upregulates the proapoptotic protein Bim, which overcomes chemotherapy resistance mechanisms^8,9^. In addition, Ishizawa et al.^10^ reported that ONC201 inhibits mammalian target of rapamycin complex 1 (mTORC1) signaling in hematological melanomas, likely through ATF4-mediated induction of the mTORC1 inhibitor DDIT4 (DNA damage inducible transcript 4). Consistent with previous studies, ONC201 was recently shown to induce ATF4 in cutaneous T-cell lymphomas, which in turn inactivates Akt as well as JAK/STAT and NF-kB pathways^11^. Independent of TRAIL-mediated proapoptotic activity, ONC201 has been reported to reduce cyclin D1 and retinoblastoma protein (pRb) expressions, and to cause cell arrest at the G1 phase of the cell cycle^12^. Recent studies have suggested a novel mechanism of action by which ONC201 targets the mitochondria^13,14^. Breast cancer cell lines treated with ONC201 showed mitochondrial structural damage, functional impairment and gene suppression^14^. Considered together, these studies indicate that ONC201 has a broad spectrum of activity that induces several antiproliferative signaling pathways depending on the cellular context and environment.

ONC201 has shown antitumor activity in vitro across multiple cancer cell types. Screening libraries of hematologic and colorectal cancer cell lines showed that the efficacy of ONC201 treatment is dose and time dependent^15^. Prabhu et al.^8^ observed significant inhibition of cell proliferation 72 h post-ONC201 application (1–10 μM treatments), this process was synchronically aligned with the induction of apoptotic markers. Similarly, when screening 23 cancer cell lines, Kline et al.^7^ observed differential responses to ONC201 treatments after 72 h: melanoma cells were most sensitive with an EC_50_ in the nanomolar range, whereas colorectal cell lines were less sensitive with an EC50 of 3–10 μM. To study the mechanistic regulation of ONC201, Allen et al.^9^ treated a colorectal cell line with at micromolar concentrations of ONC201 for 24–48 h. Interestingly, Amoroso et al.^16^ improved the efficacy of prostate cancer irradiation therapy by pretreatment with ONC201 for a period of 24 h at concentrations of 5–15 μM. The latter study suggests that short-term treatment with ONC201 is sufficient to prime cancer cells and induce pre-apoptotic phenotype without pronounced effects on cell viability. This approach would therefore facilitate combined therapeutic applications related to ONC201 and its effects on cell metabolism.

ONC201 exerts synergistic activity in combination with: (1) cytarabine or 5-azacytidine in AML cells^8^; (2) Bcl2 antagonist ABT199 in MCL-1 cells^10^; and (3) 5-fluorouracil, irinotecan, oxaliplatin or the RTK inhibitor crizotinib in the pancreatic cancer cell lines PANC-1 and HPAF-II^17^. Notably, the efficacy of ONC201 includes targeting of cancer stem cells that prime tumor initiation, relapse and metastasis^18^. ONC201 is also reported to downregulate genes associated with Wnt signaling and self-renewal in cancer stem cells from colorectal cancer, prostate cancer and glioblastoma^18^.

ONC201 treatment causes cell death by upregulating genes and proteins involved in endoplasmic reticulum (ER) stress or genes related to the integrated stress response (ISR). The only reported ISR-ONC201 responsive factors are ATF4 and CCAAT/enhancer binding protein (C/EBP) homologous protein (CHOP), which trigger the phosphorylation and activation of the eukaryotic translation initiation factor eIF2α^7,10^.

The transactivator CHOP belongs to the C/EBP family and is implicated in various stress response pathways, such as ER stress^19^, redox stress^20^, and nutrient deprivation stress^21,22^. CHOP plays key functional roles in apoptosis and autophagy^23,24^, as well as in inhibition of adipocyte differentiation^25^. CHOP expression is regulated by basic-leucine zipper (bZIP) class transcription factors; deletion mutant analysis shows that the bZIP domain plays a critical role in CHOP-induced apoptosis^26^. The 5’ flanking sequence of the *CHOP* promoter contains overlapping cis-acting CAAT enhancer binding-activating transcription factor (ATF) and cyclic AMP response element (CRE) DNA-binding elements that bind to different complexes containing C/EBPβ, ATF2, ATF3, and ATF4 in various cell types^27^. CHOP is ubiquitously expressed at very low levels; however, pathological conditions that induce overwhelming ER stress upregulate CHOP expression, resulting in apoptosis primarily regulated by upstream factors such as protein kinase RNA-like endoplasmic reticulum kinase (PERK), activating transcription factor 6 (ATF6), and inositol requiring protein 1 (IRE1)^28^. Notably, a recent study showed that treatment of a metastatic prostate cancer cell line (PC3) with ONC201 could induce the expression of ATF4, ATF6 and IRE1-XBP1 signaling, upstream of CHOP^16^. Despite an abundance of research on ONC201, however, the mechanism of action by which it induces ISR proteins in cancer cells has yet to be determined.

The molecular pathways regulated by ONC201 are well documented but the transcriptional changes in response to ONC201 treatment are not well defined, particularly in the context of metastatic and nonmetastatic cancers. Here, we aimed to identify the differentially expressed genes and associated signaling pathways associated with CHOP expression in colorectal cancer cell lines with or without ONC201 treatment. To this end, we used SW480 and LS-174T as cell models for nonmetastatic and metastatic colorectal cancer cells, respectively. In response to ONC201 treatment, CHOP expression was upregulated in both cell lines; however, a complex process of CHOP regulation was observed in the metastatic cell line. Furthermore, posttranscriptional regulation of CHOP by alternative splicing was significantly altered in response to ONC201 treatment.

## Results

### ONC201 induces apoptosis in the human colorectal cancer cell lines

**Figure 1:**
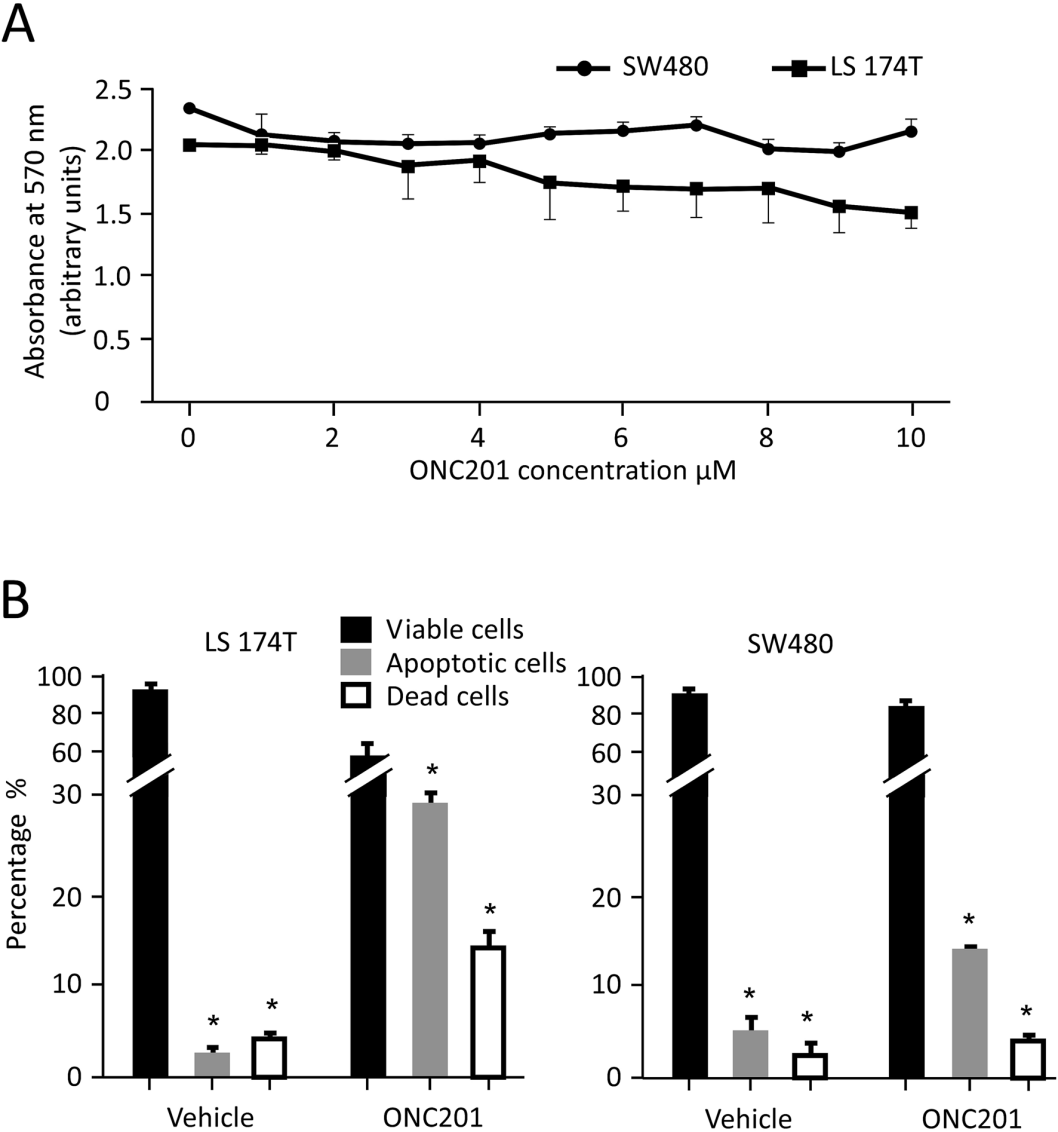
Response of Dukes' type B colorectal adenocarcinoma cell lines to ONC201 treatments. (**A**) Dose-dependent response of metastatic LS-174T and nonmetastatic SW480 cells to ONC201 treatment. Cells were plated in a 96-well plates, cell viability was assayed by MTT and detected using spectrophotometer. (**B**) Flow cytometric analysis of LS-174T and SW480 cells treated with or without 10 μM ONC201 for 48 h. Apoptotic cells were detected using annexin V kit, values are expressed as mean ± SD. Significant values were set as *P < 0.01 (n = 3 each in duplicates).

Previous studies have shown that ONC201 has an antimetastatic effect^29^. To determine whether metastatic (LS-174T) and nonmetastatic (SW480) Dukes’ type B colorectal adenocarcinoma cell lines were responsive to ONC201 treatment, we treated these cells with increasing concentrations of ONC201 or with vehicle as a control. As shown in Fig. 1A, the nonmetastatic cell line SW480 was resistant to ONC201 toxicity and its proliferation rate was relatively sustained, independent of drug concentration. On the other hand, metastatic LS-174T cell proliferation/viability was gradually reduced in response to increasing concentrations of ONC201. These data suggest that ONC201 has a dose-dependent growth inhibitory effect on metastatic cells and only a moderate growth inhibitory effect on nonmetastatic cells.

To gain a better understanding of the cytotoxic effect of ONC201 on these cell lines, we used flow cytometry to evaluate cell proliferation stages in response to ONC201 treatment. As indicated in Fig. 1B, flow cytometric analyses were in agreement with cytotoxicity test results. Compared to nonmetastatic SW480 cells, which showed an 81% ± 1.5% survival rate, the viability of metastatic LS-174T cells was significantly reduced, i.e., 57.3% ± 1.8%. Relative to the vehicle-treated control, LS-174T cells treated with ONC201 showed a significant, tenfold increase in apoptotic cells and 3.3-fold increase in cell death. In contrast, nonmetastatic SW480 cells were more resistant to the drug treatment, with apoptotic and dead cells increasing by only 2.0–2.5-fold relative to the control (Fig. 1B).

### Pathway enrichment of differential microarray-based gene expression

**Figure 2:**
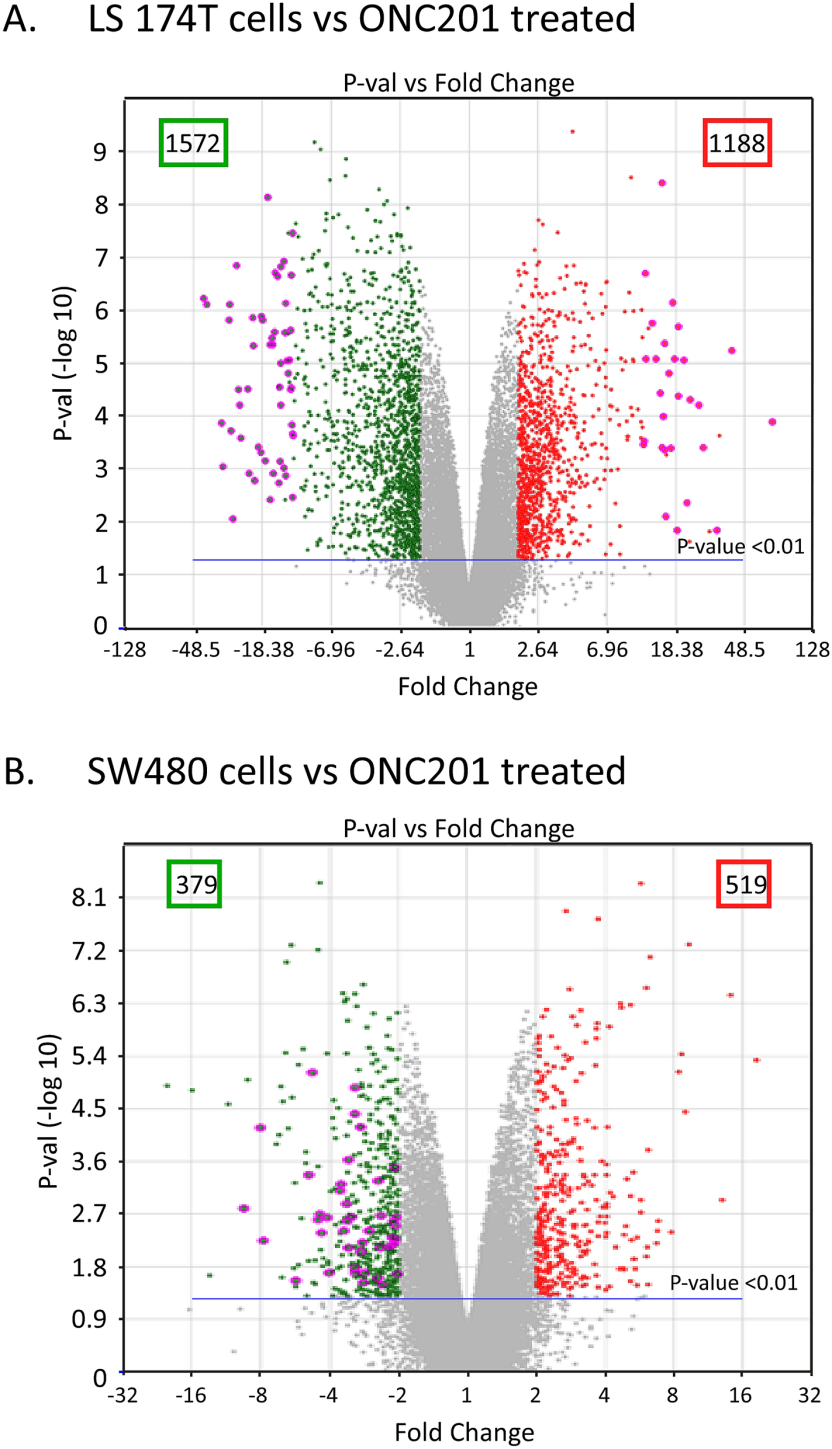
Affymetrix microarray analysis of LS-174T and SW480 cells treated with or without ONC201. Volcano graphs illustrating the differential gene expression in LS174T (**A**) and SW480 (**B**) cells in response to ONC201 treatment. Each dot represents one gene that had detectable expression in either cell line in response to ONC201 treatment. The horizontal line marks the threshold (*P* < 0.01). The color code defines an upregulated gene as red and a downregulated gene as green, with the change ≥ twofold relative to control (vehicle-treated cells). The software Affymetrix Expression Console (Version 1.0) was used for the analysis as described by the manufacturer (Material and Methods).

The studied colorectal cancer cell lines exhibited a differential response to ONC201 treatment, suggesting a unique mechanism of action that may be related to the metastatic transformation of LS-174T cells. Gaining an understanding of these mechanisms will provide new insights into the effectiveness of ONC201 treatment. To this end, we performed microarray transcriptome profiling of RNA samples from LS-174T and SW480 cells with or without ONC201 treatment; we used Affymetrix expression console software for data analysis, applying a fold-change difference ≥ 2 and a *p*-values < 0.01 to determine which genes were differentially expressed between ONC201- and vehicle-treated cells. This critical differentially expressed transcript filter is shown in volcano plots in Fig. 2A,B. In total, we detected 1,188 and 1,572 upregulated and downregulated gene transcripts, respectively, in ONC201-treated metastatic LS-174T cells relative to the expression in vehicle-treated cells (Fig. 2A). In comparison, reduced numbers of differentially regulated transcripts were observed in nonmetastatic SW480 cells post-ONC201 treatment, i.e., only 519 and 379 gene transcripts were upregulated and downregulated, respectively, (Fig. 2B).

Next, we performed Gene Ontology (GO) and Kyoto Encyclopedia of Genes and Genomes (KEGG) pathway enrichment analyses on the differentially regulated transcripts. For both cell lines, the top signaling pathways that showed statistically significant regulation (*p* ≤ 0.001) in response to ONC201 treatment are listed in Supplemental Tables 1 and 2. Initially, these pathways were classified into major network mechanisms including oncogenesis, cell cycle, cellular metabolic pathways, DNA repair, micro-RNAs, and stress; the latter was affiliated only with ONC201-treated nonmetastatic SW480 cells (Supplemental Table 2). In comparison, the overall number of regulated signaling pathways and associated genes in ONC201-treated metastatic LS-174T cells was higher than that observed in treated nonmetastatic SW480 cells.

Detailed analysis of the total gene expression profile associated with each signaling pathway revealed remarkable diversity between the metastatic and nonmetastatic cancer cell lines. In drug-treated LS-174T cells, we observed a notable global downregulation of genes associated with oncogenesis, cell cycle, and DNA repair networks. Whereas, cell homeostasis networks, such as cellular metabolic pathways and micro-RNAs, showed a comparable number of upregulated or downregulated genes (Supplemental Table 1). Surprisingly, ONC201-treated SW480 cells showed fewer regulated genes that were almost equivalently upregulated or downregulated, at least in part, for the studied networks. Notably, a large number of stress response network genes were upregulated only in the ONC201-treated nonmetastatic SW480 cells (Supplemental Table 2).

### Meta-analysis of differentially regulated pathways and genes in metastatic versus nonmetastatic cells in response to ONC201 treatment

In response to ONC201 treatment, observed differences between the two cell lines implied the existence of differentially regulated mechanisms. Accordingly, we performed a comparative meta-analysis of all the differentially expressed genes and their influence on signaling pathways. We used a computational method that considered the interplay between the gene products in the pathway in response to the drug treatment and scored a predicted functional perturbation for each protein (Supplemental Figure S1); the data were then further adjusted by Bonferroni corrections. This approach predicts functional results for the microarray data. For instance; the apoptosis map generated from extrinsic and intrinsic gene expression changes in response to ONC201 treatment does not explain the moderate apoptotic phenotype in nonmetastatic SW480 cells compared with the phenotype in metastatic LS-174T cells (Supplemental Figure S2A and S3A, respectively). On the other hand, the predicted functional perturbation changes in the apoptotic pathway clearly indicate that the observed phenotype in SW480 cells is due to a moderate induction of apoptotic genes (e.g., Casp7 and Casp9) that were not detected in the differential gene expression profile. This approach predicts the hidden functional effects of altered upstream regulatory genes (Supplemental Figure S1B and S3B). This map also shows that, upon ONC201 treatment, only the intrinsic apoptotic pathway is affected in nonmetastatic SW480 cells whereas both the extrinsic and intrinsic apoptotic pathway effectors are increased in metastatic LS-174T cells; hence, LS-174T cells show a higher apoptotic fraction (see Fig. 1).

**Figure 3:**
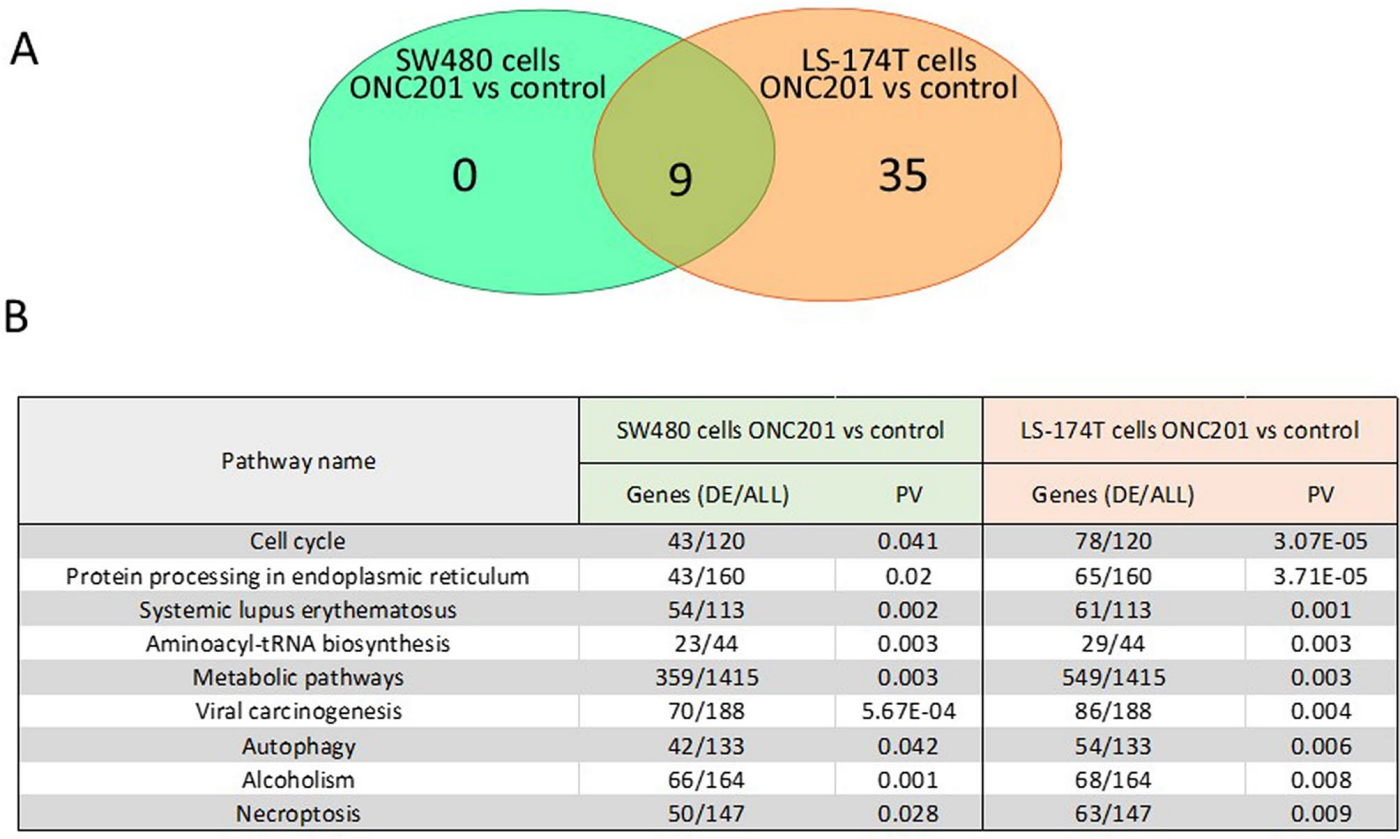
Meta-analysis alignment of differentially expressed common pathways in response to ONC201 treatment. (**A**) Venn diagram illustrating the number of signaling pathways differentially perturbed in response to ONC201 treatment in nonmetastatic and metastatic cell lines. The overlapping area indicates the number of signaling pathways commonly altered in both cell types. (**B**) Nine common pathways modified in both cell types in response to ONC201 treatment and the number of altered genes per pathway.

Interestingly, when the computational methods were applied to both cell types, nine pathways were significantly perturbed in SW480 cells posttreatment with ONC201, whereas 44 signaling pathways were perturbed in the metastatic cell line LS-174T (Fig. 3A). Figure 3B shows the pathways that are commonly perturbed in both cell lines, while Table 1 details the 35 pathways that are perturbed only in LS-174T.

Detailed analysis of the *p*-values revealed that ONC201 treatment profoundly influenced genes associated with cell cycle signaling and ER- processing proteins, especially in metastatic LS-174T cells. Among the cell cycle regulatory genes, 36% and 65% were respectively perturbed in SW480 and LS-174T cells (Fig. 3B). Likewise, ONC201 administration altered 27% and 41% of genes regulating ER function in SW480 and LS-174T cells, respectively (Fig. 3B). Similarly, the other seven signaling pathways were also differentially regulated in either cell type in response to the drug treatment; of particular interest, genes associated with metabolic pathways, autophagy, and necroptosis may explain the observed phenotype shown in Fig. 1.

As previously mentioned, 35 signaling pathways were significantly perturbed in the genes differentially regulated in metastatic LS-174T cells but not nonmetastatic SW480 cells (Table 1). Of particular interest, changes to gene expression in the cellular senescence and colorectal cancer signaling pathways were most pronounced with notably low *p*-values. In addition, p53 signaling, DNA replication, and other metabolic signaling pathways were significantly decreased but to a lesser extent than the earlier described pathways.

**Table 1:** 35 signaling pathways with significantly differentially regulated genes were observed in the metastatic LS-174T cell line. (Differential Expression/All gene detected, DE/ALL), *p*-value (PV).

Pathway name	SW480 cells ONC201 verus control		LS-174T cells ONC201 versus control	
	Genes (DE/All)	PV	Genes (DE/All)	PV
Small cell lung cancer	28/91	0.886	45/91	3.79E−06
Fanconi anemia pathway	13/48	1	34/48	1.36E−05
Cellular senescence	48/154	0.457	73/154	2.03E−04
Shigellosis	62/224	0.145	87/224	2.64E−04
Homologous recombination	Nov-39	1.000	28/39	5.05E−04
Salmonella infection	66/242	1.000	97/242	8.87E−04
Platinum drug resistance	20/69	1.000	37/69	9.84E−04
Colorectal cancer	25/86	0.405	44/86	9.90E−04
Human T-cell leukemia virus 1 infection	62/215	0.437	96/215	0.001
P53 signaling pathway	27/73	0.114	42/73	0.002
Oocyte meiosis	34/117	1.000	47/117	0.002
Nucleotide excision repair	Nov-42	1.000	26/42	0.003
Mismatch repair	Aug-23	1.000	18/23	0.003
Biosynthesis of cofactors	40/142	1.000	67/142	0.003
Valine, leucine, and isoleucine degradation	15/47	1.000	29/47	0.003
Carbon metabolism	36/118	1.000	58/118	0.003
Biosynthesis of amino acid	22/73	1.000	42/73	0.003
DNA replication	Nov-36	1.000	31/36	0.003
Apoptosis	42/134	0.500	62/134	0.003
FoxO signalling pathway	36/127	1.000	57/127	0.004
Hepatitis C	44/155	1.000	64/155	0.005
Fatty acid degradation	Dec-42	1.000	25/42	0.006
Base excision repair	Oct-33	1.000	21/33	0.008
Pathogenic Escherichia coli infection	44/189	1.000	70/189	0.009
Inositol phosphate metabolism	17/72	1.000	36/72	− 0.0011
RNA transport	38/172	1.000	70/172	0.0013
Fatty acid metabolism	14/54	1.000	29/54	0.0015
Pyruvate metabolism	Nov-39	1.000	23/39	0.0016
AMPK signalling pathway	37/118	0.538	51/118	0.0019
Pyrimidine metabolism	14/52	1.000	28/52	0.0019
MAPK signalling pathway	72/287	1.000	99/287	0.0023
Fluid shear stress and atherosclerosis	34/132	1.000	56/132	0.0028
Amyotrophic sclerosis	81/334	1.000	115/334	0.0036
Glycine, serine and threonine metabolism	Nov-38	1.000	22/38	0.0037
Glucagon signalling pathway	27/103	1.000	45/103	0.0047

**Figure 4:**
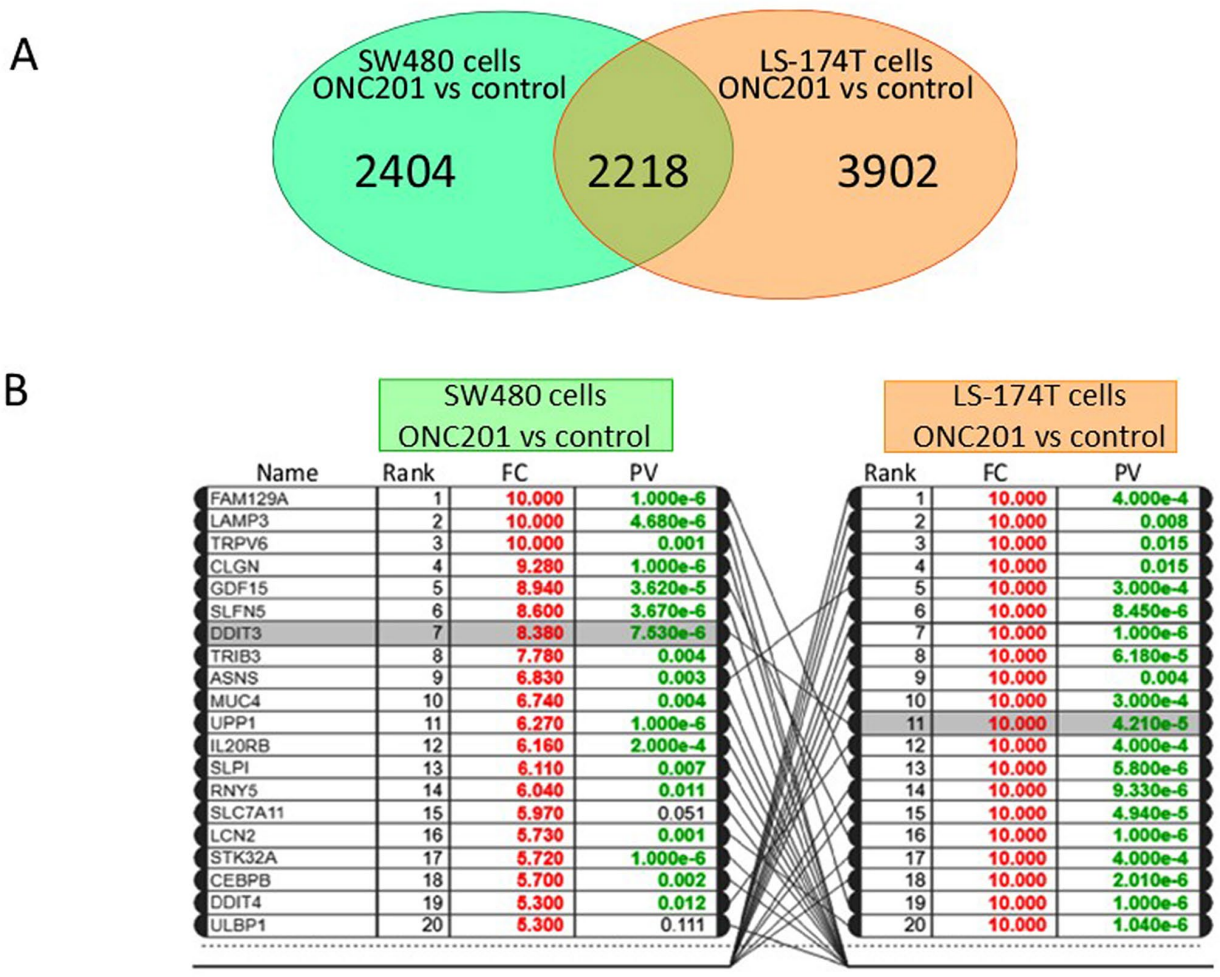
Meta-analysis Alignment of the differentially expressed common genes in response to ONC201 treatment. (**A**) A Venn diagram illustrating the total number of genes perturbed in response to ONC201 treatment in the nonmetastatic and metastatic cell lines. The overlapping areas indicate the numbers of genes that are commonly altered in both cell types. (**B**) Top ranked transcripts differentially regulated in LS-174T and SW480 cells in response to ONC201 treatment are shown, with significantly low *p*-values in green and fold expression in red color.

We also performed comparative analysis of all data to identify genes associated with the differentially regulated pathways and subsequently ranked these genes in accordance with their *p*-values (Fig. 4A). Data analysis revealed that 2,404 and 3,902 genes were differentially regulated in the nonmetastatic SW480 and metastatic LS-174T cell lines, respectively. Of these, 2,218 were found to be commonly impaired in both cell types, albeit with varying *p*-values (Fig. 4A). The top 15 genes that were significantly upregulated in either cell line in response to ONC201 are listed in Fig. 4B. Notably, FAM129A, DDIT3/CHOP, and ASNS gene transcripts were significantly upregulated in both cell lines (*p* ≤ 0.001). FAM129A (also known as NIBAN1 or Niban apoptosis regulator 1) encodes a protein that is highly expressed in cancer. Since the cell lines are carcinogenic in nature, such a transcript should have been detected in our microarray data. DDIT3/CHOP is a transcription factor and a member of the C/EBP family. ASNS (asparagine synthetase) is involved in asparagine synthesis and facilitates progression through G1 phase of the cell cycle (Fig. 4B).

**Figure 5:**
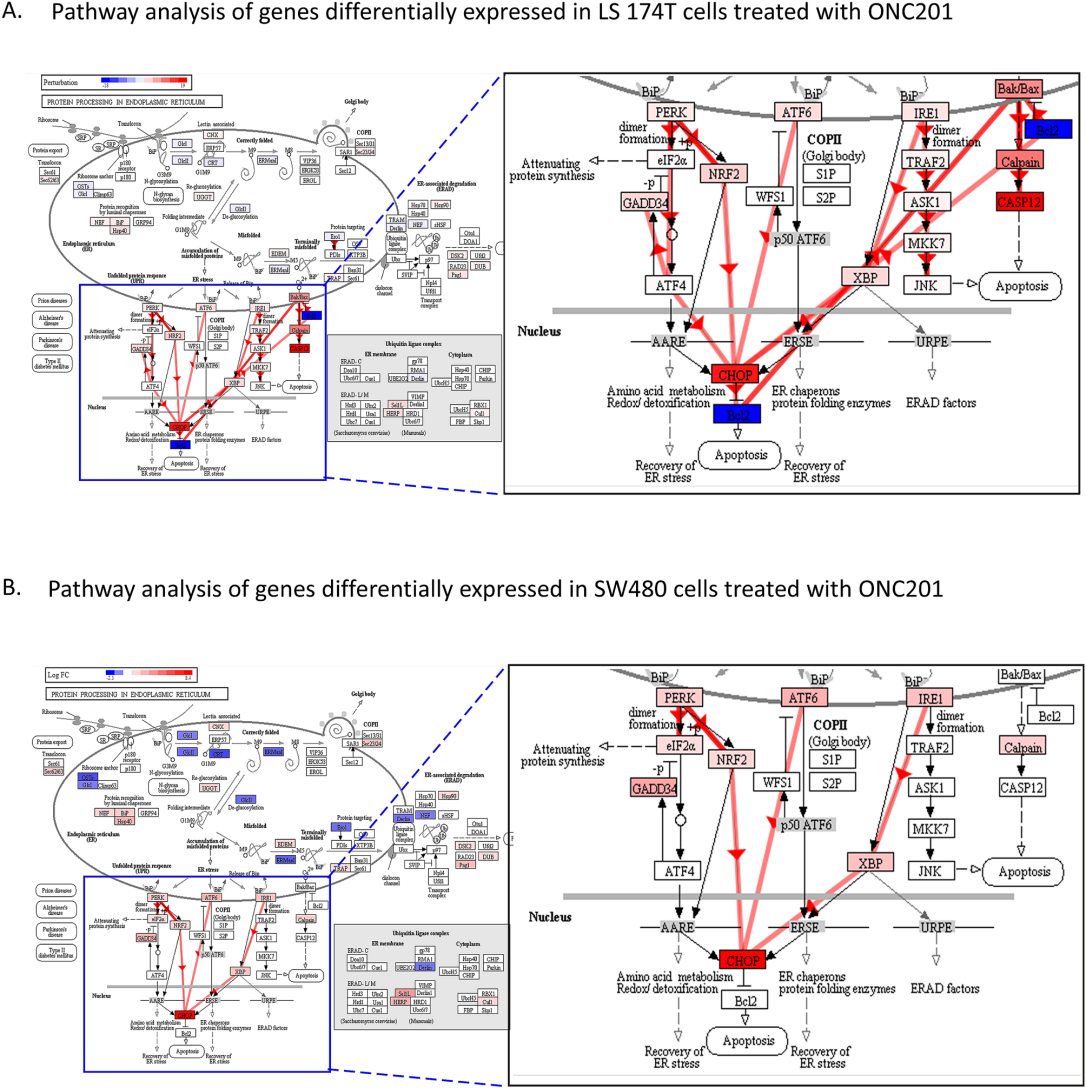
Schematic showing CHOP regulation in response to ONC201 treatment. (**A**) Pathway analysis of genes differentially expressed in metastatic colorectal cancer LS174T cells treated with ONC201. (**B)** Pathway analysis of genes differentially expressed in nonmetastatic colorectal cancer SW480 cells treated with ONC201.

Our initial results indicated that ONC201 induces apoptosis differently in both cell lines and since CHOP is a critical regulatory factor for this pathway therefore, we focused our study on the regulatory mechanisms associated with this gene. Mapping the differentially expressed gene transcripts detected in microarray data onto the map of the ER protein processing pathway^30,31^ revealed similar, but nonidentical, regulatory mechanisms upstream of CHOP for each treated cell line (Fig. 5). In metastatic LS-174T cells, ONC201 treatment induced upregulation of CHOP transcripts through the upregulation of IRE1, ATF6, PERK, and Bak/Bax signaling networks (Fig. 5A). These regulatory mechanisms were also observed in ONC201-treated SW480 cells, except for Bak/Bax pathway expression (Fig. 5B). In addition, transcripts of downstream antiapoptotic BCL2 were significantly downregulated in metastatic LS-174T cells, relative to their expression in nonmetastatic SW480 cells, post-ONC201 treatment.

### ONC201 treatment elicits apoptosis in LS-174T and SW480 human colorectal cancer cells

**Figure 6:**
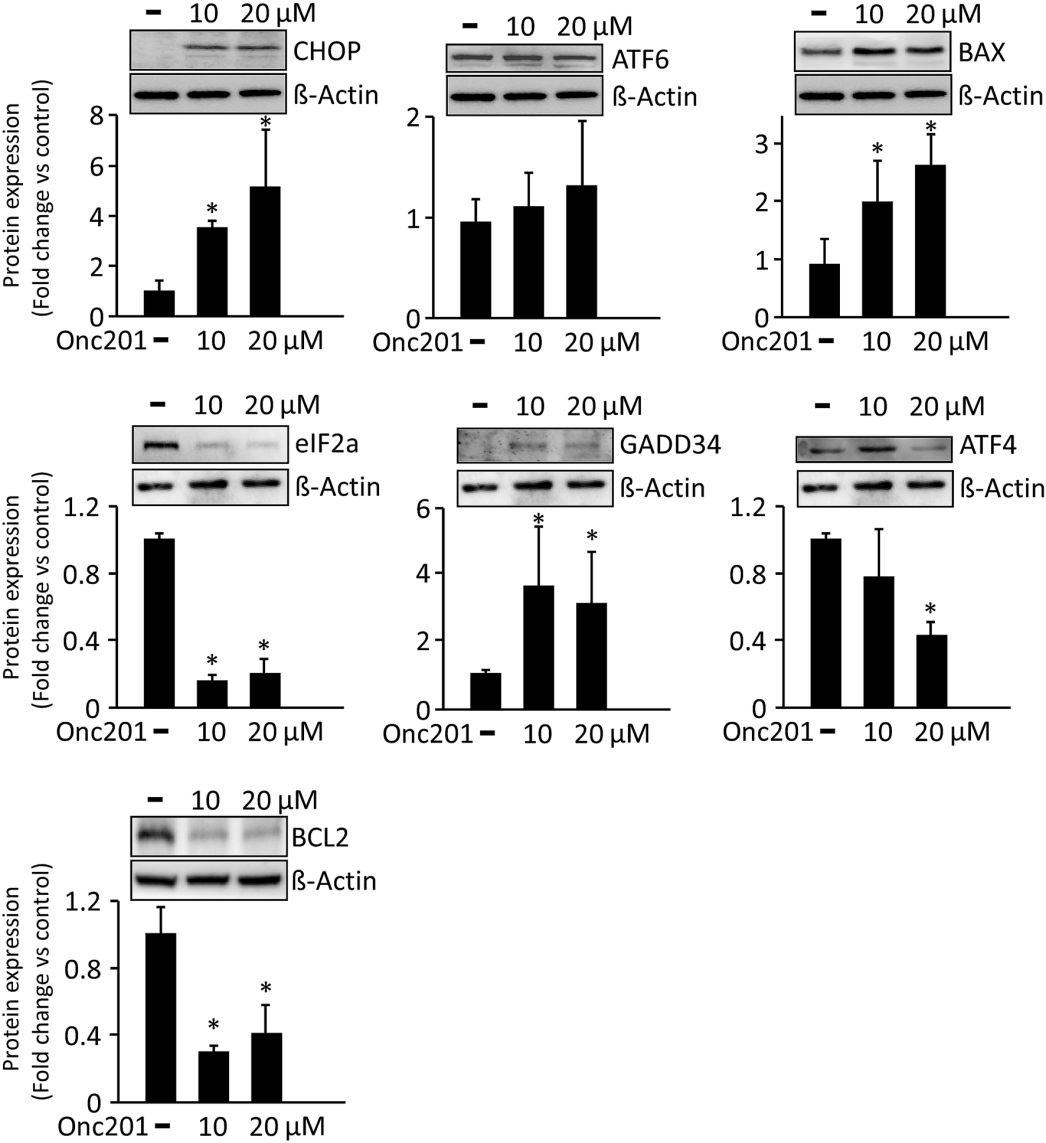
Western bolt analysis of metastatic LS-174T cells treated with or without ONC201, at varying concentrations. Differential expression patterns of different proteins that are involved in CHOP regulation and ER stress are shown. Densitometric analysis of the representative Western blots is shown (mean ± SD), with respect to housekeeping gene β-actin in different treatment groups (n = 3). Significant values were set as *P < 0.01. The software ImageJ V1.49O (https://imagej.nih.gov/ij/) was used to quantify the immunoblot signals as a mean of the grey/white scale of all the pixels in a band.

**Figure 7:**
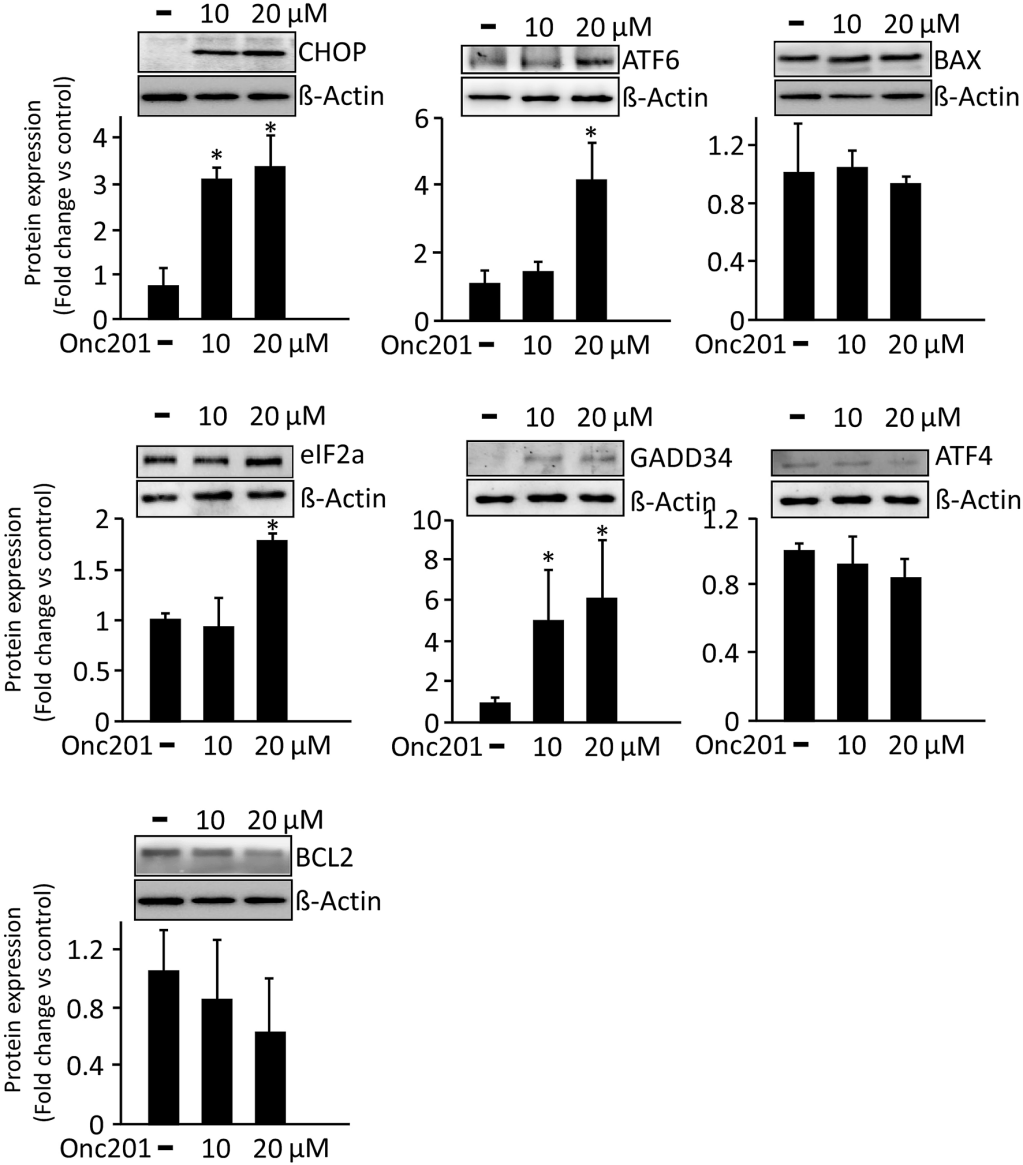
Western blot analysis of nonmetastatic SW480 cells treated with or without ONC201, at varying concentrations. Differential expression patterns of different proteins involved in CHOP regulation and ER stress are shown. Densitometric analysis of the representative Western blots is shown (mean ± SD), with respect to housekeeping gene β-actin in different treatment groups (n = 3). Significant values were set as *P < 0.01. The software ImageJ V1.49O (https://imagej.nih.gov/ij/) was used to quantify the immunoblot signals as a mean of the grey/white scale of all the pixels in a band.

The microarray gene expression data were validated by western blot analysis for selected proteins associated with the upregulation of CHOP. Indeed, treatment with ONC201 resulted in a significant increase in CHOP protein expression in both LS-174T (Fig. 6) and SW480 (Fig. 7) cells. The upstream CHOP regulatory proteins associated with different ER signaling pathways were also studied and showed similar patterns of expression to those observed in the microarray study. Specifically, ATF6 protein expression increased in response to ONC201 treatments in both cell lines, and the response was statistically significant when nonmetastatic SW480 cells were treated with 20-µM ONC201. In addition, Bax proteins were significantly increased in metastatic LS-174T cells, but not in nonmetastatic SW480 cells, post-ONC201 treatments (Figs. 5 and 7). PERK-regulated proteins (eIF2a, GADD34, and ATF4) were differentially expressed in response to ONC201 treatment in these cell lines. EIF2a protein expression was significantly increased in nonmetastatic SW480 cells (Fig. 7) while being significantly reduced in metastatic LS-174T cells at 20-µM ONC201 (Figs. 5). Low ONC201 concentration treatments were associated with slight reductions in ATF4 expression in both cell lines; however, at high ONC201 concentrations (i.e., 20 µM), significantly reduced ATF4 expression was observed in metastatic LS-174T cells only (Fig. 6). In contrast, GADD34 expression was significantly augmented in both cell lines in response to ONC201 treatment (Figs. 6 and 7; Supplemental Figures S4 and S5). Microarray meta-analysis indicated a significant reduction in *BCL2* transcripts, particularly in ONC201-responsive metastatic LS-174T cells; this was confirmed at the protein level at which BCL2 protein was significantly downregulated post-ONC201 treatments (Fig. 6). Contrastingly, BCL2 protein expression was sustained in the presence or absence of ONC201 treatment in nonmetastatic SW480 cells (Fig. 7). Thus, microarray analysis data and protein expression levels of the studied signaling network markers were found to be alignment after ONC201 treatments.

### Alternative splicing CHOP mRNA

**Figure 8:**
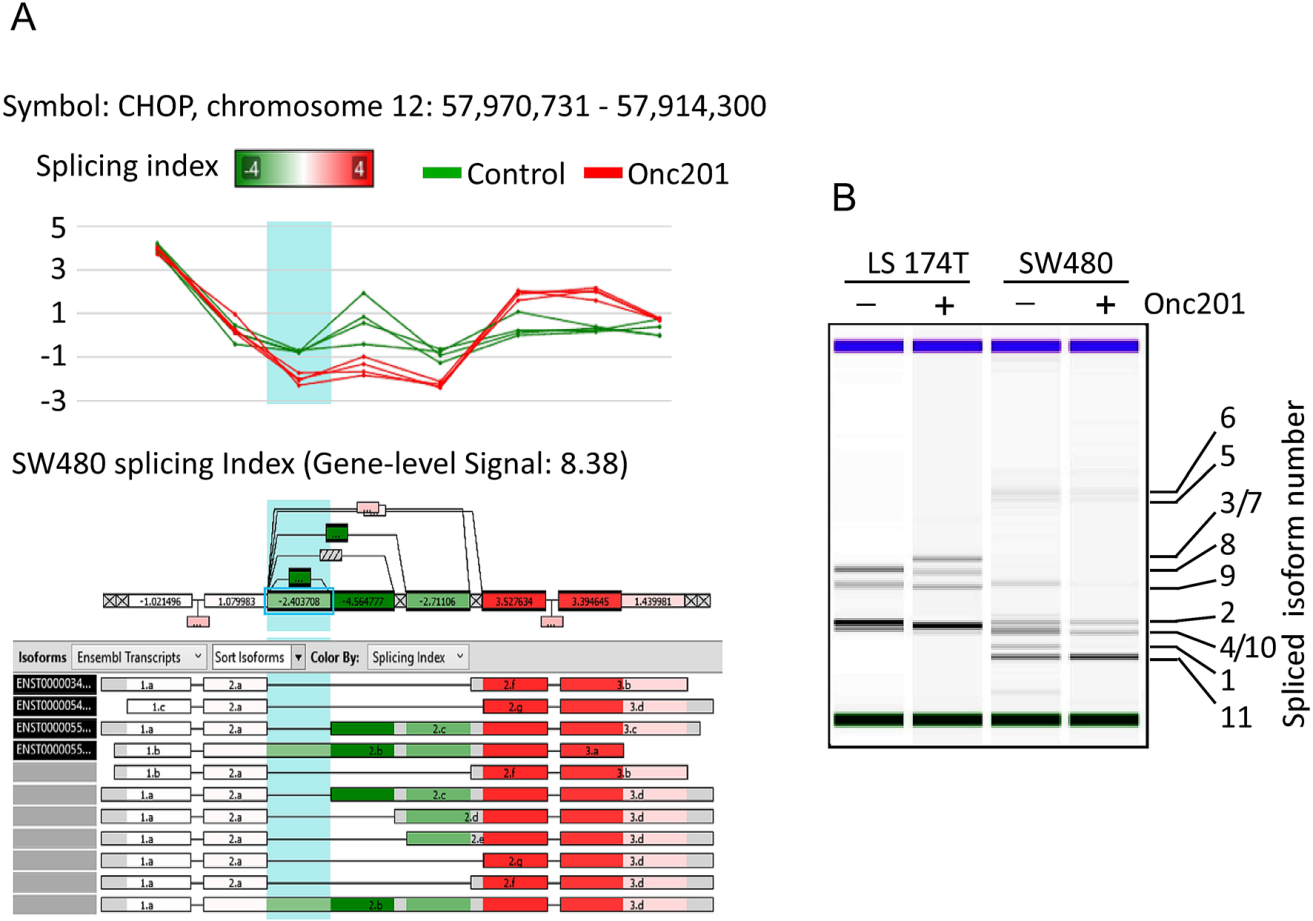
Alternative spliced variants of CHOP. (**A**) A computational analysis detecting CHOP differential alternative splicing events obtained from the microarray study of SW480 treated with and without ONC201. (**B**) Bioanalyzer electrophoresis after quantitative PCR analysis of LS-174T and SW480 cells treated with and without ONC201. CHOP was amplified using specific primers flanking the exons 1 and 4. PCR products were fractionated on Bioanalyzer microgel. Alternative spliced isoforms were mapped according to their fragment size.

Defects in RNA alternative splicing are a hallmark of cancerous cells. Many RNA splicing regulators have been studied as tumor suppressors or are associated with drug resistance^32–34^. Exon splicing analysis of CHOP showed significant variation derived from metastatic LS-174T cells treated with vehicle versus those treated with ONC201: significantly reduced (up to 4.65-fold) splicing index signal levels were found in exon 2 in samples treated with the drug (Fig. 8A). To confirm these data experimentally, we performed quantitative RT-PCR and fractionated the products on a bioanalyzer. As shown in Fig. 8B, differential splicing patterns of CHOP mRNA were observed in metastatic LS-174T and nonmetastatic SW480 cells, and these patterns were further modified in response to ONC201 treatment. This is an additional indication of the multifaceted mechanisms of action of ONC201 as an anticancer drug.

## Discussion

Using nonmetastatic SW480 and metastatic LS-174T colorectal cancer cell lines, we identified signaling pathways that were differently perturbed when cells were treated with ONC 201. In metastatic LS-174T cells, we identified differential mechanisms of CHOP regulation in response to ONC201 treatment that downregulate BCL2 specifically and induce apoptosis. Previous research has shown that ONC201 treatment interrupts ER homeostasis and induces the expression of three ER stress response signaling networks, namely PERK, ATF6, and IRE1, known to trigger unfolded protein response signaling^35,36^. Here, we report the prospective role of the ER stress Bak/Bax network in targeting CHOP regulation in the metastatic colorectal cell line LS-174T after ONC201 treatment. Additionally, our microarray data indicated that prospective crosstalk occurs between the IRE1 and Bak/Bax signaling pathways in LS-174T cells. Furthermore, the complexity of the CHOP regulatory mechanism in the metastatic cell line and the subsequent downregulation of BCL2 may explain the observed proliferation arrest and high apoptosis rates in ONC201-treated LS-174T cells relative to the response in nonmetastatic SW480 cells. Since BCL2, which was consistently downregulated in metastatic LS-174T cells, is inhibitory effector of the Bak/Bax signaling pathway^37^, we cannot exclude the prospective feedback regulatory interplay among Bax/CHOP/BCL2 as a regulatory factor in the response of LS-174T cells to ONC201 application. On the other hand, our meta-analysis indicated that the transcripts of BH3 proteins, Bak/Bax activators, are significantly upregulated, particularly in LS-174T cells; nevertheless, Bak/Bax autoactivation can occur, independently of the activator BH3s (i.e., BIM, BID, PUMA, and NOXA) following BCL2 downregulation^38^. Our observations are in accordance with previous studies that highlighted the role played by ONC201 in mediating the ER stress response in breast cancer cells^12,39^ and high-grade central nervous system glioblastoma^40^. However, in these studies, the observed ER stress response was primarily due to ATF4 activation. Lev et al.^17^ compared ONC201-sensitive HAPF-II against resistant PANC-1 pancreatic cell lines and reported a discrepancy in the ER stress response: ONC201 mediated upregulation of three ER stress response signaling molecules in PANC-1 cells, whereas ATF4 was the only protein to be upregulated in HPAF-II cells, in which substantial expression of IRE1 or ATF6 proteins was not detected. In nonmetastatic SW480 cells, ER homeostasis is restored by the upregulation of eIF2a, which explains the observed moderate effect of ONC201 treatment. Active eIF2a attenuates protein synthesis and reduces protein-processing workload on the stressed ER^41–43^. Taken together, these findings suggest that the cellular response to ONC201 treatment is cell type-dependent, but that the overall mechanisms are associated with ER stress and unfolded protein response signaling.

Gene expression profiles in colorectal cancer cells revealed that ONC201 downregulates genes associated with energy metabolism. Specifically, ONC201 reduced the gene expression of citrate carrier (SLC25A1) and fumarate hydratase (FH) that regulate the mitochondrial metabolite carrier and substrate metabolism, respectively. SLC25A1 is involved in citrate mitochondria/cytoplasm translocations for cellular energy homeostasis^44^, whereas FH plays an important role in the Krebs cycle by providing FADH and NADH to the electron transport chain for ATP production^45^. Thus, ONC201 is involved in reducing metabolic pathways that may cause energy stress in cancer cells. Similarly, Ishida et al.^46^ observed a decrease in the ATP levels associated with low glycolysis and oxidative phosphorylation that caused energy stress to cancer cells. ONC201 can also reduce mitochondrial respiration in breast cancer cells, which may lead to energy stress (reducing ATP) and result in apoptosis^14^.

In our analysis, we identified several spliced variants of CHOP that were differentially expressed in ONC201-treated metastatic and nonmetastatic colorectal cell lines. Notably, exon 2 was found to be the target for the CHOP splicing mechanism. The function of exon 2 in CHOP is unclear, although this exon encodes part of the 5’-untranslated region and in general, untranslated regions are considered to regulate the protein translation activity or mRNA expression^47,48^. CHOP belongs to the C/EBP family of transcription factors and functions as a dominant-negative inhibitor by forming heterodimers with other C/EBP members. Alternative splicing has also been reported for other family members; The C/EBP epsilon gene is regulated by an alternative translational initiation site and splicing mechanisms^49^, which generate four different isoforms with different functions^50,51^. In addition to alternative translational initiation, the expression of four alternative C/EBPε isoforms (p32, p30, p27, and p14) has been attributed to differential promoter usage and alternative splicing^51^.

In summary, the efficacy and outcome of cancer treatment is dependent on the stage of the disease. Differences between nonmetastatic and metastatic cancer cells are associated with cellular plasticity and metabolic reprogramming^52^, which lead to differential responses to chemotherapy as observed here and in other studies. In the present study, we delineated the interactive signaling mechanisms and associated genes that differentially regulate CHOP expression in nonmetastatic and metastatic colorectal adenocarcinoma cells. In ONC201-treated metastatic LS174T cells, these mechanisms lead to increased expression of the core regulators, Bak and Bax, of the intrinsic apoptosis pathway.

We acknowledge that the current study has limitations; further mechanistic studies will be required to delineate the functional role of the signaling pathways up- and downstream of CHOP in both metastatic and nonmetastatic cell types. In particular, the crosstalk among Bak/Bcl2/CHOP must be assessed. In addition, further studies are required to determine the role of the observed CHOP alternative transcripts splicing between cell lines and the observed changes in response to ONC201 treatment which could be indicative of the role by which cell responses to ONC201-mediated cell death are modulated. WE used SW480 and LS-174T as model cell lines, but it will be important to validate our observations using a variety of metastatic and nonmetastatic colorectal cell lines as well as patient-specific colorectal cells.

## Materials and methods

### Cancer cell lines and tissue culture

Dukes’ type B colorectal adenocarcinoma cell lines LS-174T (ATCC CL-188) and SW480 (ATCC CCL-228) were purchased from the American Type Culture Collection (ATCC, Manassas, VA, USA). Cells were grown in RPMI1640 media (Invitrogen Corporation, Carlsbad, CA, USA) supplemented with 10% fetal bovine serum (Invitrogen Corporation) and 100 U/mL of penicillin–streptomycin (Gibco, Carlsbad, CA, USA) and incubated at 37 °C in 5% CO_2_. ONC201 (SML1068, Sigma Aldrich, Germany) was dissolved in dimethyl sulfoxide (DMSO, D8418, Sigma Aldrich, Germany) to a stock concentration of 100-mM and stored at − 20 °C in aliquots. At the time of the experiments, 100-mM ONC201 solution was further diluted to generate stock solution of 10- or 20-mM in 10% DMSO. The control (vehicle) used was 0.01% DMSO. Cells were treated with ONC201 at concentrations of 10- or 20-µM for 24 h before experiments were initiated while controls were treated with vehicle (i.e., 0.01% DMSO).

### Preparation of protein extract and western blot analysis

Cells were harvested and lysed using modified RIPA buffer (50 mM Tris–HCl at pH 7.5, 150-mM NaCl, 1% Triton × 100, 1-mM EDTA, 0.5% sodium deoxycholate and 0.1% SDS). Cell lysates were quantified using a Pierce BCA Protein Assay Kit (Thermo Fisher Scientific GmbH, Driesch, Germany) and equal amounts of protein (30 μg) were resolved on 8%–12% polyacrylamide gels before using transferred to polyvinylidene fluoride membranes (EMD Millipore Corporation, Billerica, MA, USA) as previously described^53^. After blocking, membranes were blotted with the corresponding primary and horseradish peroxidase-linked secondary antibodies^54^. The primary antibodies used were as follows: β-actin (ab8224), eIF2a (ab169528), GADD34 (ab9869) and ATF4 (ab85049) (all from Abcam, USA). CHOP (2895S), Bax D2E11 (5023T), and BCL2 D55G8 (4223T) were purchased from Cell Signaling, USA; ATF6 (ALX-804-381-C100) was purchased from ANZO, USA. Finally, immunoblots were detected by chemiluminescence using the Chemidoc MP system (Bio-Rad, USA). ImageJ V1.49O software (https://imagej.nih.gov/ij/) was used to quantify the immunoblot signals as the mean value of the gray/white scale of all pixels in a band^55^.

### MTT cytotoxicity assay

Cytotoxicity assays were performed as previously described^56^. Briefly, cells were seeded into 96-well plates at a density of 10^6^ cells/well and incubated at 37 °C in 5% CO_2_ for 12 h. The control wells were then treated with vehicle while the experimental wells were treated with increasing concentrations (1- to 10-µM) of ONC201. After 48 h, 1 mg/ml of MTT (3-[4,5-dimethylthiazol-2-yl]-2,5-diphenyltetrazolium, Promega Bio Sciences LLC, San Luis Obispo, CA, USA) was added to each well. The developed Formazan crystals were subsequently dissolved in 10% SDS/0.04 eq/L HCl solution for 1 h at 37 °C. Absorbance was measured at 490 nm using a microplate reader (DTX880; Beckman Coulter, Brea, CA, USA). Cell survival was expressed as the percentage of control cells as follows: CS (%) = (mean *A*_treated well_/mean *A*_control well_) × 100.

### Fluorescence-based flow cytometry

To quantify apoptosis, cells were stained with an annexin V kit (Abcam, Cambridge, MA, USA) according to the manufacturer’s protocol. Briefly, 1 × 10^5^ cells were treated with 10-μM of ONC201 for 48 h. Cells were collected after trypsinization and then washed twice with PBS. Cell pellets were resuspended in 100 μL of 1 × annexin-binding buffer and then 1 μL of an annexin V-fluorescein isothiocyanate (FITC) working solution was added to the 100-μL of cell suspensions. The suspensions were subsequently incubated on ice for 10 min in the dark. Cell suspension volume was brought to 250 μL with 1 × binding buffer and the stained cells were placed on a glass slide before being covered with a glass coverslip. The cells were observed under a fluorescence microscope using a filter set for FITC detection. Alternatively, the stained cells were immediately analyzed by flow cytometry FACS Calibur (BD Biosciences, Becton Dickinson, Franklin Lakes, NJ, USA). For each measurement, at least 20,000 cells were counted. After drug treatment, cells were collected, incubated with propidium iodide (PI) and Annexin V (BioLegend, USA), and analyzed by flow cytometry. The apoptosis percentage was calculated using the following formula: apoptosis (%) = PI (%) and annexin V double-positive cells with drug − PI (%) and Annexin V double-positive cells without drug).

### RNA extraction, microarray array , and PCR assays

Total RNA was isolated using the Trizol–Chloroform method as described by Al Madhoun et al.^57^. Isolated RNA was quantified and RNA integrity was assessed by microfluidic analysis using a Bioanalyser 2100 (Agilent Technologies**,** Santa Clara, CA, USA). For microarray assays, total RNA (100 ng) from each sample (in triplicate) was reverse transcribed as per the manufacturer’s protocol (GeneChip WT PLUS Reagent, Thermo Fisher Scientific). Purified cDNA was fragmented, labeled, and hybridized onto a GeneChip Human Transcriptome Array 2.0 (Thermo Fisher Scientific GmbH) for 16 h at 45 °C and 60 rpm in an Affymetrix GeneChip Hybridization Oven 640. Chips were washed and stained using an Affymetrix GeneChip Fluidics Station 450 (Thermo Fisher Scientific) and scanned with an Affymetrix GeneChip Scanner 3000 7G (Thermo Fisher Scientific). CEL data files were analyzed using Affymetrix expression console software (version 1.0) provided by the manufacturer.

For spliced isoform analysis, we followed similar procedures to those described by Al Al Madhoun et al.^32^; cDNA was synthesized from 1 µg of RNA by reverse transcription using a QuantiTect Reverse Transcription Kit (Qiagen Inc., Hilden, Germany) as previously described^58^. Reverse-transcribed RNA was used as a template for CHOP spliced isoform quantitative PCR amplification using specific primers and a FastStart SYBR Green Kit (Roche Applied Sciences, Penzberg, Germany). Forward (5′-TAAGGCACTGAGCGTATCATG-3′) and reverse (5′-CTGGACAGTGTCCCGAAGGAGAAA-3′) primers were designed using Primer Bank^59^. PCR products were run on an Agilent 2100 Bioanalyzer system using a High Sensitivity DNA Electrophoresis Kit, as instructed by the manufacturers (Agilent Technologies) and then quantified using High Sensitivity DNA assay software.

### Bioinformatics and meta-analysis of microarray data

Functional classification and enrichment analysis was performed based on GO annotation the KEGG database^30,60^. To detect pathways differentially perturbed in metastatic cancer cells relative to nonmetastatic cells following ONC201 treatment, we used a computational method to integrate differential gene expression into predefined pathways as described in Rivera et al.^61^. Briefly, we graphed P = (G, I), where P are pathways, G are their gene sets, and I are the interactions between these genes. For each cell line, we input the fold change of the treatment versus that of the control and determined which pathways were perturbed upon ONC201 treatment. The Liptak-Stouffer z-score was calculated as the perturbation of each subgraph. The most perturbed subpathway was then computed according to the algorithm of Rivera et al.^61^. We also applied Bonferroni corrections to the final computed *p*-value of the most perturbed pathways for more stringent results.

### Statistical analysis

All experiments and assays were conducted in technical duplicates or triplicates for three biological samples. Results were combined and statistical significance was determined using a two-tailed Student’s *t*-test assuming equal variance. Test were performed in GraphPad Prism version 8.0. Data are presented as means ± standard error of the mean (SEM) as previously described^62^.

## Supplementary Information

Supplementary Information.

## Data Availability

All data generated and analyzed during this study are included in this article.
