# Supplementary material for: Microarray analysis reveals ONC201 mediated differential mechanisms of CHOP gene regulation in metastatic and nonmetastatic colorectal cancer cells
Source: Sci Rep. 2021 Jun 4;11:11893. doi: 10.1038/s41598-021-91092-8 (PMC8178367; doi:10.1038/s41598-021-91092-8)
Supplement: Supplementary file 1 — Supplementary Information. [file 41598_2021_91092_MOESM1_ESM.docx]

**Microarray Analysis of Metastatic and Non-metastatic Colorectal Cancer Cells Revealed Differential Pathways Regulation in Response to ONC201 treatment.**

**Authors**

Ashraf Al Madhoun^1,2*^, Dania Haddad^1^, Mustafa Al Tarrah^1^, Sindhu Jacob^1^, Waleed Al-Ali^3^, Rasheeba Nizam^1^, Lavina Miranda^2^, Fatema Al-Rashed^4^, Sardar Sindhu^2,4^, Rasheed Ahmad^4^, Milad S. Bitar^3^, and Fahd Al-Mulla^1*^

**Affiliations**

^1^ Department of Genetics and Bioinformatics, Dasman Diabetes Institute, Dasman, 15462, Kuwait.

^2^ Department of Animal and Imaging Core Facilities, Dasman Diabetes Institute, Dasman, 15462, Kuwait.

^3^ Department of Pharmacology and Toxicology, Faculty of Medicine, Kuwait University, 046302 Jabriya, Kuwait.

^4^ Department of Immunology an Microbiology, Dasman Diabetes Institute, Dasman, 15462, Kuwait.

**Correspondence**

[fahd.almulla@dasmaninstitute.org](mailto:fahd.almulla@dasmaninstitute.org) (F.A.-M.); [ashraf.madhoun@dasmaninstitute.org](mailto:ashraf.madhoun@dasmaninstitute.org) (A.A.M.);

Tel.: +965-2224-2999 (ext. 2805) (A.A.M.); +965-6777-1040 (F.A-M.). Department of Genetics and Bioinformatics, Dasman Diabetes Institute, Dasman, 15462, Kuwait.


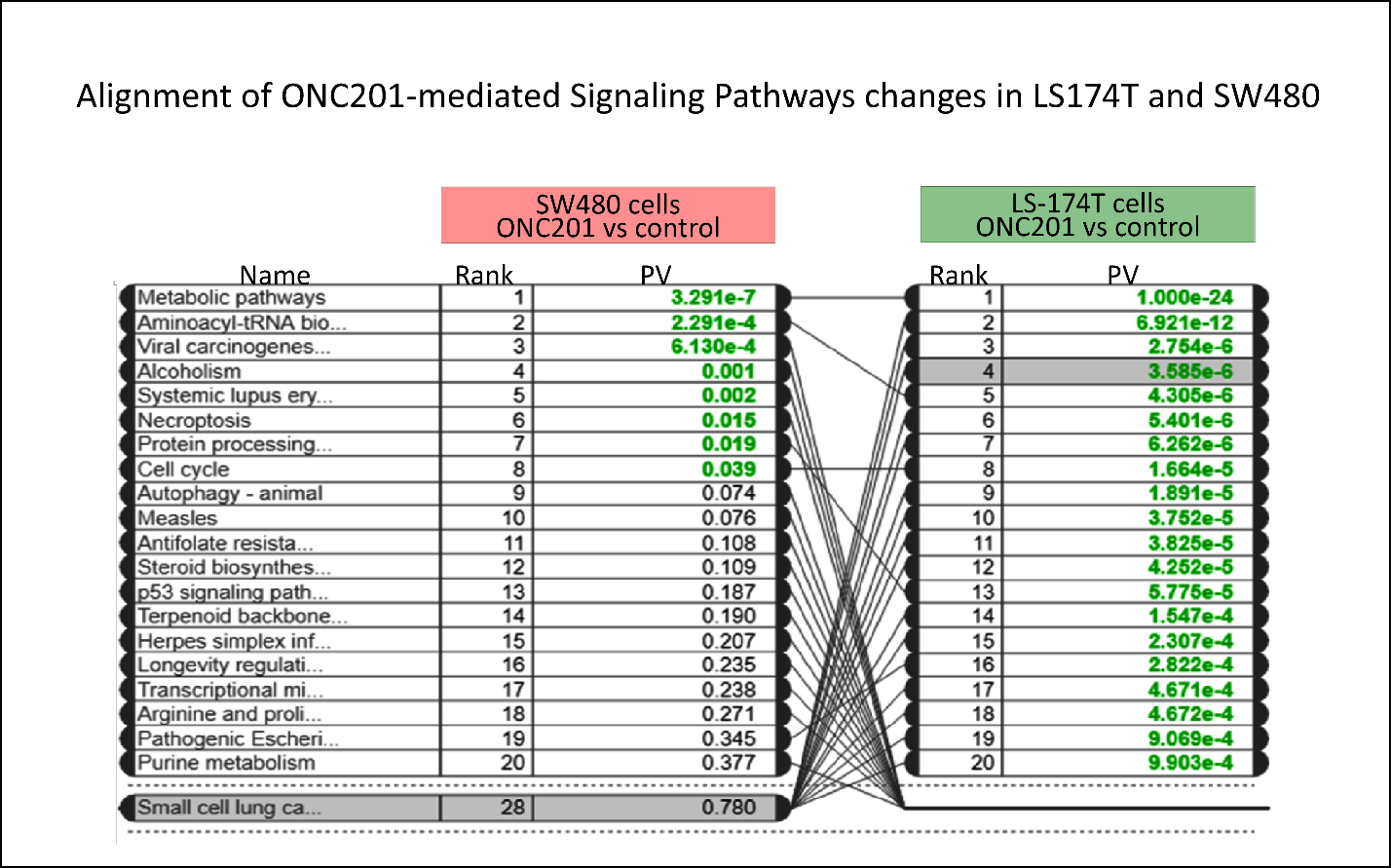


**Supplemental Figure S1: Meta-analysis Alignment of the differentially expressed common pathways and genes Before Bonferroni adjustments**. Top ranked pathways that are differentially regulated in metastatic LS-174T cells and non-metastatic SW480 cells in response to ONC201 treatment are shown, with significantly low P-values in green color.


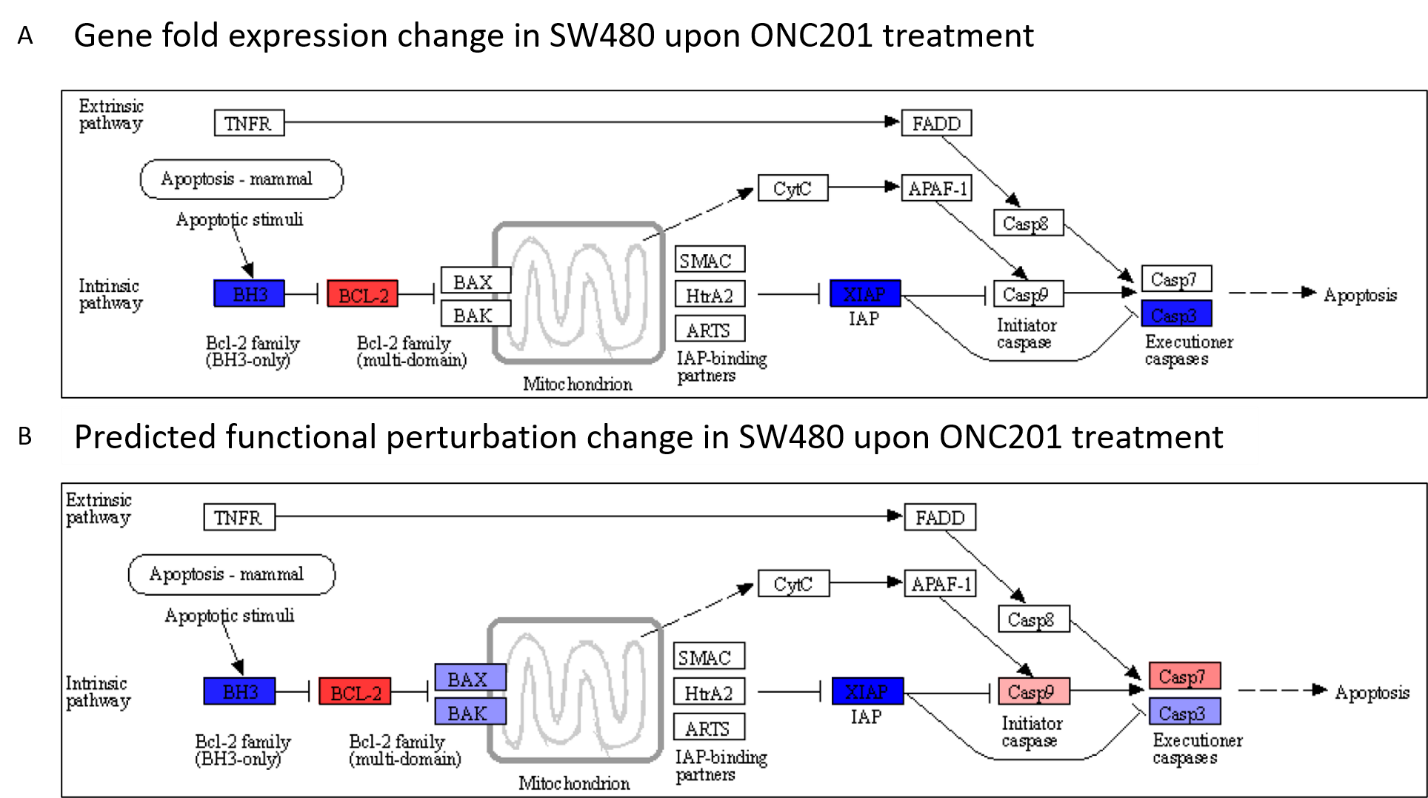
**Supplemental Figure S2: Gene expression perturbation in non-metastatic SW480 cells upon ONC201 treatment.** A) gene fold expression changes of some players in the intrinsic apoptotic pathway. Blue indicates decreased gene expression, red denotes increased expression, and white denotes unchanged expression upon ONC201 treatment; B) Predicted functional perturbation in non-metastatic SW480 cells upon ONC201 treatment. Blue indicates decreased gene expression, red denotes increased expression, light blue indicates moderate decreased gene expression, pink denotes moderate increased expression and white denotes unchanged expression.


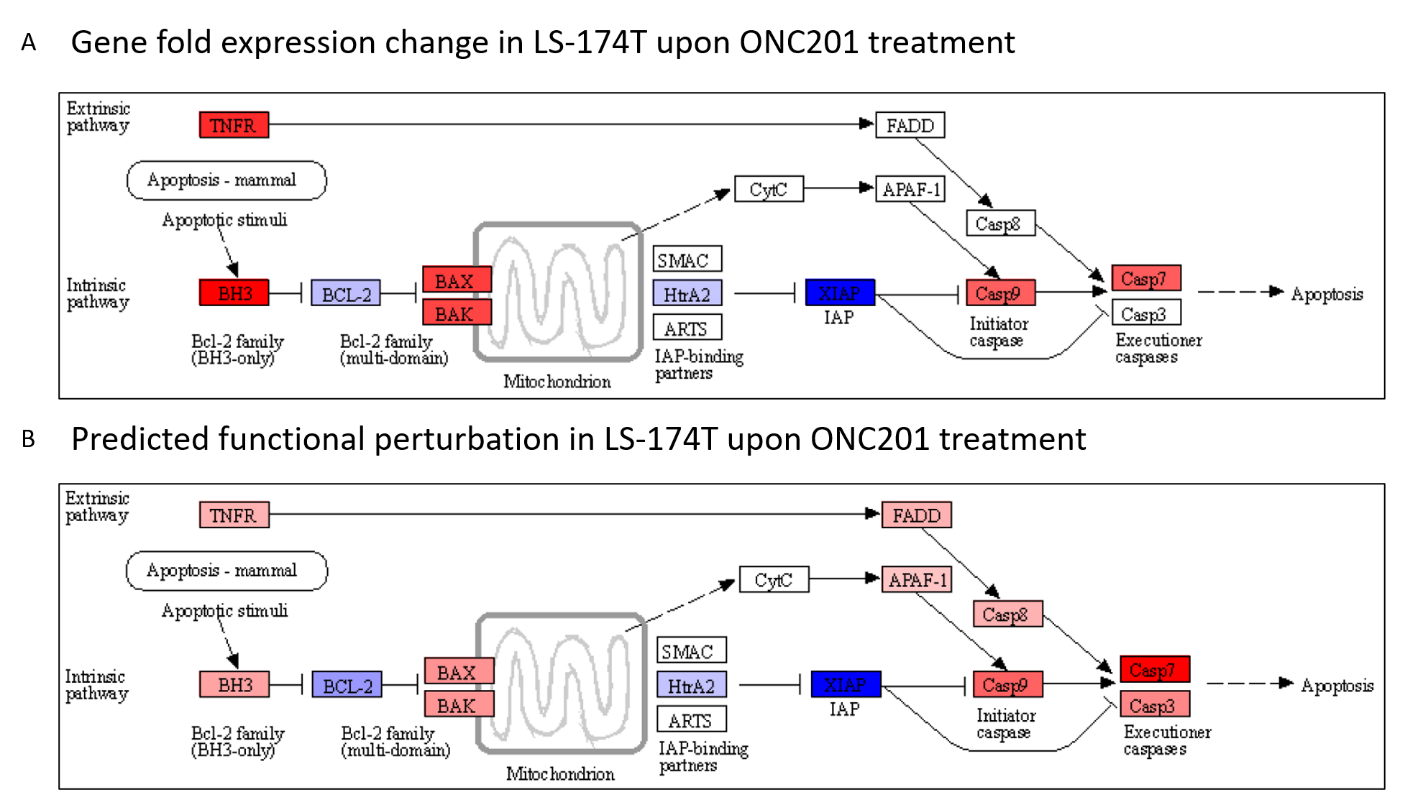
**Supplemental Figure S3: Gene expression perturbation in metastatic LS-174T cells upon ONC201 treatment.** A) gene fold expression changes of some players in the extrinsic and the intrinsic apoptotic pathway. Blue indicates decreased gene expression, red denotes increased expression, and white denotes unchanged expression upon ONC201 treatment; B) Predicted functional perturbation in metastatic LS-174T cells upon ONC201 treatment. Blue indicates decreased gene expression, red denotes increased expression, light blue indicates moderate decreased gene expression, pink denotes moderate increased expression and white denotes unchanged expression.

Supplemental Figure S4


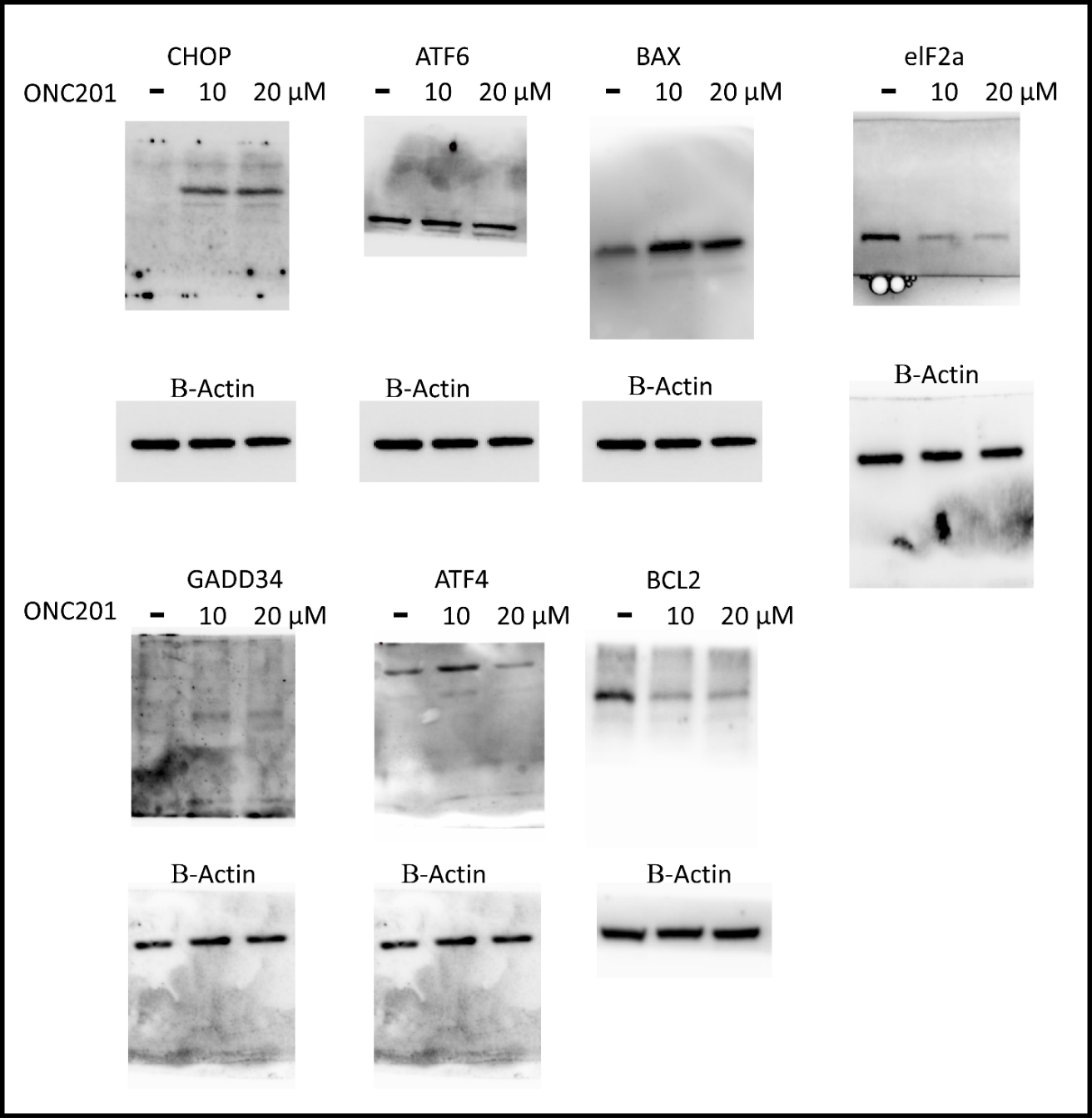


**Supplemental Figure S4: Western bolt analysis of metastatic LS-174T cells treated with or without ONC201, at varying concentrations**. Protein extracts from LS-174T cells were fractionated on 12% polyacrylamide gel and transferred into nitrocellulose membranes. After blotting the membranes were sliced and incubated with the appreciated antibodies based on the protein prospective molecular weight. Then, the membranes were put in the ChemiDoc system for imaging after incubation with ECL. The observed images are representative western blots of three independent experiments. housekeeping gene β-actin was also blotted. Some blots were stripped and re-blotted with β-actin antibodies.

Supplemental Figure S5


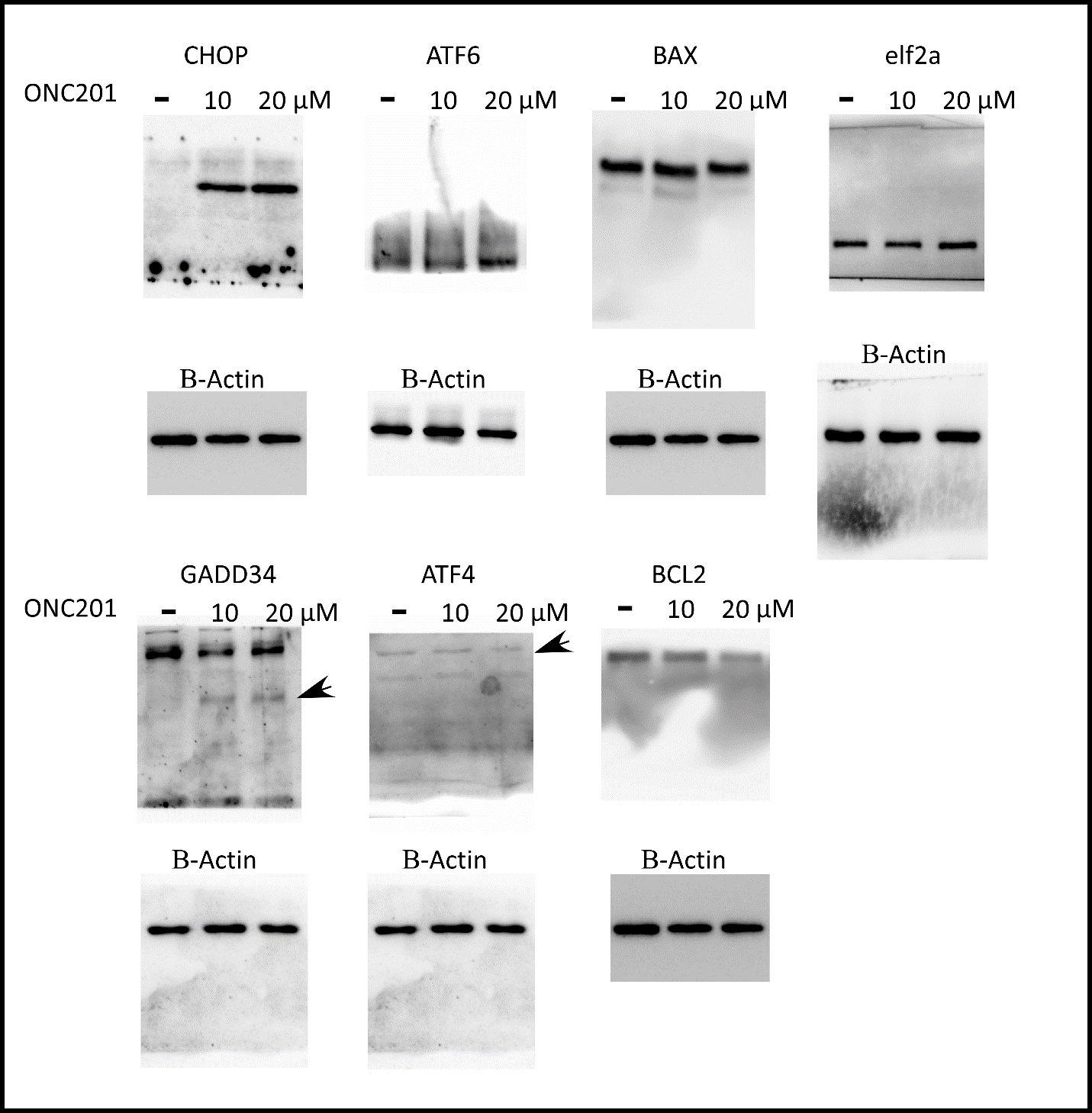


**Supplemental Figure S5: Western bolt analysis of non-metastatic SW480 cells treated with or without ONC201, at varying concentrations**. Protein extracts from SW480 cells were fractionated on 12% polyacrylamide gel and transferred into nitrocellulose membranes. After blotting the membranes were sliced and incubated with the appreciated antibodies based on the protein prospective molecular weight. Then, the membranes were put in the ChemiDoc system for imaging after incubation with ECL. The observed images are representative western blots of three independent experiments. housekeeping gene β-actin was also blotted. Some blots were stripped and re-blotted with β-actin antibodies.


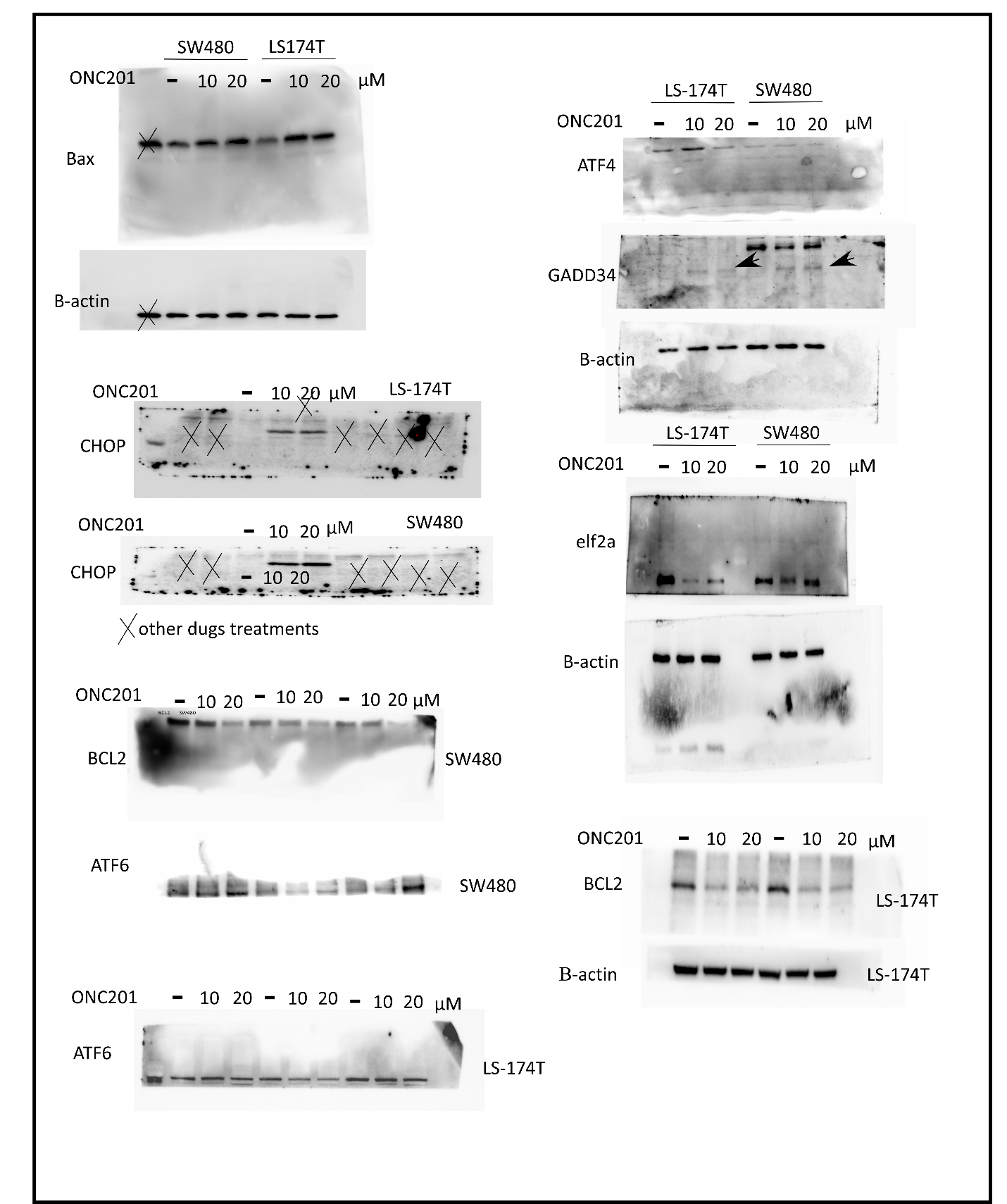


**Supplemental Figure S6:** Original blots for western. Wells that were not taken into consideration due to an unused control or unused ONC201 concentration were crossed out.

Table 1 : Top 5 gene networks differentially expressed in the Dukes' type B colorectal adenocarcinoma metastatic LS-174T cell line treated with ONC201

| **Associated Network Functions** | **Total # of Genes** | **# of Upregulated Genes** | **# of Downregulated Genes** | **Significance** | **P-value** |
| --- | --- | --- | --- | --- | --- |
| **Oncogenes** | | | | | |
| Retinoblastoma (RB) in Cancer | 57 | 1 | 56 | 43.82 | 0 |
| Integrated Breast Cancer Pathway | 24 | 7 | 17 | 3.36 | 0.000438 |
| Gastric Cancer Network 1 | 15 | 1 | 14 | 10.19 | 0 |
| Gastric Cancer Network 2 | 13 | 1 | 12 | 7.31 | 0 |
| Pathways Affected in Adenoid Cystic Carcinoma | 15 | 5 | 10 | 4.12 | 0.000076 |
| Hepatitis C and Hepatocellular Carcinoma | 11 | 4 | 7 | 2.94 | 0.001159 |
| Signaling Pathways in Glioblastoma | 13 | 7 | 6 | 2.46 | 0.003503 |
| Pathways in clear cell renal cell carcinoma | 13 | 8 | 5 | 2.14 | 0.007276 |
| Imatinib and Chronic Myeloid Leukemia | 6 | 3 | 3 | 2.71 | 0.001937 |
| Apoptosis-related network due to altered Notch3 in ovarian cancer | 12 | 9 | 3 | 3.83 | 0.000147 |
| Imatinib and Chronic Myeloid Leukemia | 6 | 3 | 3 | 2.71 | 0.001937 |
| **Cell Cycle** | | | | | |
| Cell Cycle | 35 | 0 | 35 | 15.56 | 0 |
| G1 to S cell cycle control | 27 | 2 | 25 | 14.02 | 0 |
| Cell Cycle Checkpoints | 10 | 1 | 9 | 2.16 | 0.006869 |
| HIV Life Cycle | 5 | 1 | 4 | 2.37 | 0.00431 |
| Regulation of sister chromatid separation at the metaphase-anaphase transition | 9 | 0 | 9 | 7.07 | 0 |
| Circadian rhythm related genes | 24 | 12 | 12 | 2.18 | 0.006581 |
| Metapathway biotransformation Phase I and II | 24 | 14 | 10 | 2.73 | 0.001841 |
| Mitotic Metaphase and Anaphase | 5 | 0 | 5 | 2.65 | 0.002233 |
| **Cellular Metabolic pathways** | | | | | |
| Pyrimidine metabolism | 25 | 2 | 23 | 8.35 | 0 |
| PI3K-Akt Signaling Pathway | 36 | 22 | 14 | 2.24 | 0.005776 |
| Amino Acid metabolism | 20 | 8 | 12 | 5.54 | 0.000003 |
| Cholesterol Biosynthesis | 11 | 0 | 11 | 9.94 | 0 |
| Cholesterol biosynthesis | 11 | 0 | 11 | 6.49 | 0 |
| PPAR signaling pathway | 13 | 4 | 9 | 3.33 | 0.00047 |
| Vitamin D Receptor Pathway | 22 | 14 | 8 | 2.13 | 0.007471 |
| Adipogenesis | 19 | 12 | 7 | 2.93 | 0.001179 |
| Nucleotide Metabolism | 8 | 1 | 7 | 4.84 | 0.000014 |
| Androgen receptor signaling pathway | 13 | 7 | 6 | 2.1 | 0.007981 |
| One Carbon Metabolism | 9 | 4 | 5 | 4.27 | 0.000053 |
| Fatty Acid Biosynthesis | 6 | 1 | 5 | 2.6 | 0.002515 |
| Exercise-induced Circadian Regulation | 10 | 6 | 4 | 2.98 | 0.001038 |
| Copper homeostasis | 10 | 6 | 4 | 2.52 | 0.003042 |
| GPCR ligand binding | 7 | 3 | 4 | 11.17 | 0 |
| SREBF and miR33 in cholesterol and lipid homeostasis | 6 | 2 | 4 | 3.1 | 0.000787 |
| TCA Cycle and Deficiency of Pyruvate Dehydrogenase complex (PDHc) | 5 | 1 | 4 | 2.52 | 0.00302 |
| **DNA repair** | | | | | |
| Histone Modifications | 19 | 0 | 19 | 7.37 | 0 |
| DNA Damage Response | 20 | 4 | 16 | 8.03 | 0 |
| DNA IR-Double Strand Breaks (DSBs) and cellular response via ATM | 12 | 0 | 12 | 3.44 | 0.000361 |
| ATM Signaling Pathway | 11 | 1 | 10 | 4.28 | 0.000053 |
| Mismatch repair | 5 | 0 | 5 | 3.89 | 0.000129 |
| Homologous recombination | 6 | 1 | 5 | 4.01 | 0.000097 |
| **Micro RNAs** | | | | | |
| miR-targeted genes in lymphocytes - TarBase | 62 | 23 | 39 | 5.47 | 0.000003 |
| miR-targeted genes in muscle cell - TarBase | 53 | 23 | 30 | 5.17 | 0.000007 |
| miR-targeted genes in epithelium - TarBase | 46 | 20 | 26 | 4.91 | 0.000012 |
| miRNA Regulation of DNA Damage Response | 21 | 4 | 17 | 5.83 | 0.000001 |

Table 2 Top 6 gene networks differentially expressed in the Dukes' type B colorectal adenocarcinoma non-metastatic SW480 cell line treated with ONC201

| **Associated Network Functions** | **Total # of Genes** | **# of Upregulated Genes** | **# of Downregulated Genes** | **Significance** | **P-value** |
| --- | --- | --- | --- | --- | --- |
| **Oncogenes** | | | | | |
| Oncostatin M Signaling Pathway | 6 | 3 | 3 | 2.53 | 0.002919 |
| Liver steatosis AOP | 8 | 5 | 3 | 2.74 | 0.001828 |
| TCA Cycle Nutrient Utilization and Invasiveness of Ovarian Cancer | 2 | 2 | 0 | 2.36 | 0.00441 |
| **Cell Cycle** | | | | | |
| Cell Cycle | 7 | 0 | 7 | 2.14 | 0.007198 |
| Hair Follicle Development: Induction (Part 1 of 3) | 5 | 1 | 4 | 2.26 | 0.005514 |
| Nuclear Receptors Meta-Pathway | 17 | 6 | 11 | 3.18 | 0.00066 |
| Regulation of sister chromatid separation at the metaphase-anaphase transition | 3 | 0 | 3 | 2.43 | 0.003697 |
| **Cellular Metabolic pathways** | | | | | |
| Cholesterol Biosynthesis | 5 | 0 | 5 | 4.95 | 0.000011 |
| Cholesterol biosynthesis | 6 | 0 | 6 | 4.82 | 0.000015 |
| Benzo(a)pyrene metabolism | 4 | 4 | 0 | 4.61 | 0.000024 |
| Sterol Regulatory Element-Binding Proteins (SREBP) signaling | 9 | 3 | 6 | 4.58 | 0.000026 |
| Transcriptional cascade regulating adipogenesis | 4 | 4 | 0 | 3.89 | 0.000129 |
| Mevalonate pathway | 3 | 0 | 3 | 3.49 | 0.000323 |
| SREBF and miR33 in cholesterol and lipid homeostasis | 4 | 0 | 4 | 3.3 | 0.000507 |
| Amino Acid metabolism | 8 | 7 | 1 | 3.02 | 0.000951 |
| Folate Metabolism | 6 | 2 | 4 | 2.5 | 0.003149 |
| Vitamin B12 Metabolism | 5 | 1 | 4 | 2.29 | 0.005081 |
| Gap junction trafficking and regulation | 4 | 1 | 3 | 2.06 | 0.008717 |
| **DNA repair** | | | | | |
| Histone Modifications | 20 | 0 | 20 | 17.15 | 0 |
| **Micro RNAs** | | | | | |
| miR-targeted genes in epithelium - TarBase | 26 | 13 | 13 | 7.22 | 0 |
| miR-targeted genes in muscle cell - TarBase | 25 | 11 | 14 | 5.28 | 0.000005 |
| miR-targeted genes in lymphocytes - TarBase | 28 | 13 | 15 | 5.22 | 0.000006 |
| miR-targeted genes in leukocytes - TarBase | 13 | 5 | 8 | 4.24 | 0.000058 |
| **Stress** | | | | | |
| ATF4 activates genes | 4 | 3 | 1 | 2.33 | 0.004698 |
| Photodynamic therapy-induced unfolded protein response | 10 | 9 | 1 | 9.93 | 0 |
| Exercise-induced Circadian Regulation | 6 | 5 | 1 | 3.26 | 0.000545 |
| White fat cell differentiation | 4 | 3 | 1 | 2.33 | 0.004698 |
|  |  |  |  |  |  |
| VEGFA-VEGFR2 Signaling Pathway | 12 | 8 | 4 | 2.24 | 0.005807 |
| EGF/EGFR Signaling Pathway | 11 | 6 | 5 | 3.1 | 0.000787 |
